# A 3D In Vitro Model of the Human Hepatobiliary Junction

**DOI:** 10.1002/advs.202514855

**Published:** 2026-04-20

**Authors:** Ashley D. Westerfield, Katarzyna A. Grzelak, Katie Katsuyama, Vardhman Kumar, Bess M. Miller, Joa Yun, Jesse Kirkpatrick, David Mankus, Margaret E. Bisher, Abigail K.R. Lytton‐Jean, Z. Gordon Jiang, David D. Lee, Christopher S. Chen, Sangeeta N. Bhatia

**Affiliations:** ^1^ Institute of Medical Engineering and Science Massachusetts Institute of Technology Cambridge Massachusetts USA; ^2^ David H. Koch Institute for Integrative Cancer Research Massachusetts Institute of Technology Cambridge Massachusetts USA; ^3^ Department of Biological Engineering Massachusetts Institute of Technology Cambridge Massachusetts USA; ^4^ Department of Medicine Brigham and Women's Hospital Boston Massachusetts USA; ^5^ Department of Medicine Beth Israel Deaconess Medical Center Boston Massachusetts USA; ^6^ Department of Surgery Beth Israel Deaconess Medical Center Boston Massachusetts USA; ^7^ Department of Biomedical Engineering and the Biological Design Center Boston University Boston Massachusetts USA; ^8^ Wyss Institute for Biologically Inspired Engineering at Harvard University Boston Massachusetts USA; ^9^ Department of Electrical Engineering and Computer Science Massachusetts Institute of Technology Cambridge Massachusetts USA; ^10^ Broad Institute of MIT and Harvard Cambridge Massachusetts USA; ^11^ Howard Hughes Medical Institute Chevy Chase Maryland USA

**Keywords:** bile, bile duct engineering, hepatobiliary organoid, liver tissue engineering, organoid

## Abstract

Bile flow is an essential feature of the liver, and disruption of this process contributes to a range of liver pathologies. Efficient bile transport requires coordinated organization between hepatocytes and cholangiocytes at the hepatobiliary junction, a structure that remains poorly captured in existing in vitro models of liver disease. Here, we present a 3D multicellular spheroid‐based model of the human hepatobiliary junction. Building on advances in organoid and spheroid engineering, we co‐aggregate human hepatocytes and intrahepatic cholangiocytes, supported by murine fibroblasts, into adult hepatobiliary organoids (aHBOs). aHBOs directionally transport bile from hepatocyte canaliculi to cholangiocyte‐lined ductule‐like structures, visualized through a high‐throughput imaging assay. Hepatobiliary junction formation and bile flow dynamics are quantified over time using a fluorescent bile acid analog and AI‐assisted image analysis. When subjected to hypoxia‐reoxygenation, aHBOs exhibit disrupted bile transport and distinct cell‐type‐specific responses, enabling interrogation of hepatocyte and cholangiocyte vulnerability to transplant‐associated biliary hypoxia. Our findings suggest a reversible reduction in hepatocyte canalicular function under hypoxia, followed by selective cholangiocyte death upon reoxygenation, potentially contributing to biliary dysfunction after ischemic injury. This human‐derived, scalable platform provides a phenotypically relevant model for dissecting mechanisms of biliary dysfunction and discovering therapeutics for hypoxic liver injury and cholestatic diseases.

## Graphical abstract

A 3D human organoid platform reconstructs the hepatobiliary junction between primary adult hepatocytes and intrahepatic cholangiocytes. These adult hepatobiliary organoids (aHBOs) support directional bile transport from canaliculi to ductule‐like structures, enable quantitative imaging of junction dynamics, and reveal cell‐type‐specific vulnerabilities to hypoxia‐reoxygenation relevant to biliary injury and cholestatic disease.

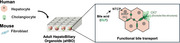

## 1 Introduction

The liver has a highly organized tissue ultrastructure that allows it to perform over 500 different vital functions, of which a crucial function is bile transport [1]. Cholestatic diseases, a class of disorders in which this function is disrupted, account for 14.2% of patients who receive a liver transplant, which is often their only curative treatment option [2]. Because of the shortage of available donor livers, recent progress has been made toward engineering liver tissues that can serve as a replacement or bridge to liver transplant. However, a therapeutic liver graft capable of bile transport requires structural and functional interaction between the two main parenchymal cell types of the liver: hepatocytes and bile duct epithelial cells, also called cholangiocytes.

Both cell types establish and maintain cell polarity, in which the apical side is exposed to bile components that are excreted into the digestive tract, while the basolateral side is exposed to nutrients and oxygen in sinusoidal circulation. Hepatocytes polarize into multipolar cells, in which their apical surfaces wrap around the cells like belts [3]. These surfaces, called the bile canaliculi, form a “chicken‐wire” network passing between cells within the liver parenchyma, into which active transport of bile acids takes place [3]. Cholangiocytes polarize into monopolar cells that are more typical of simple cuboidal epithelial cells, with one side housing the apical membrane and the other side the basal membrane. These cells line hierarchically larger branching ductal structures of the intrahepatic and extrahepatic bile ducts that form the biliary tree. Bile flows through this biliary tree and exits the liver via the common bile duct. Bile is stored in the gallbladder and later released into the small intestine to aid in the digestion of fats and lipids. Despite their differences in polarity, the apical surfaces of hepatocytes and cholangiocytes form a contiguous connection or hepatobiliary junction with each other in order to drain bile out of the liver, referred to as the Canal of Hering. The Canals of Hering functionally connect hepatocyte bile canaliculi to cholangiocyte‐lined bile ducts, forming the tiniest ductule structures of the biliary tree. The maintenance of this architecture is crucial to healthy bile synthesis and transport, as a disruption in polarity of either cell type within this junction can lead to cholestatic diseases [4, 5]. Despite the physiological importance of hepatobiliary flow in the Canals of Hering, these features have historically been difficult to characterize in native tissue [6, 7] and as a result, difficult to recapitulate in a 3D engineered tissue system.

Recent advancements in organoid technology have enabled the culture and expansion of human hepatocyte organoids, derived from induced pluripotent stem cells [8, 9, 10], fetal human liver [11], and cryopreserved primary human hepatocytes [12], [13]. Within many of these systems, hepatocytes polarize to transport bile into canaliculi, but the bile is unable to drain into any bile duct structures, as in the native liver, due to a lack of integration with cholangiocytes. At the same time, numerous developments have been made toward culturing human cholangiocytes in 3D organoid systems [14, 15, 16, 17, 18]. While these cholangiocyte organoids express a biliary gene expression profile and functional phenotype, the ability of these cells to transport and modify bile cannot be properly studied without the synthesis and secretion of bile acids from hepatocytes to cholangiocyte‐lined lumina. While coculture systems using rat and mouse cell lines have been developed to replicate this junction [19, 20, 21], no study has yet assembled the building blocks of mature human hepatocytes and cholangiocytes into a functional human hepatobiliary junction.

In this paper, we leverage both organoid and spheroid technologies to generate adult hepatobiliary organoids (aHBOs), which recreate the minimal functional unit of the human hepatobiliary junction. We scale this process up using a high‐throughput aggregation‐based culture platform, which enables parallel self‐assembly of a large number of aHBOs. Using a fluorescently labeled bile acid and an imaging assay, we visualize and quantify bile transport from bile canaliculi to cholangiocyte‐lined ductule‐like structures over time and in various culture conditions. Furthermore, we demonstrate the utility of this platform at modeling biliary pathology; specifically, we apply a hypoxia‐reoxygenation perturbation to examine how hepatocytes and cholangiocytes within a functional hepatobiliary junction respond to hypoxia. When conditioned with a hypoxia‐reoxygenation scheme that resembles the timeline observed during a clinical liver transplant, aHBOs exhibit a dysfunction in bile flow that is reminiscent of ischemic injury of the bile duct. Being the first system to recapitulate physiological and pathological bile flow within adult human cells in a 3D context, this hepatobiliary organoid model provides a platform to study the functional consequences of hypoxia on hepatobiliary function, which may improve our understanding of related clinical pathologies, including post‐transplant ischemic injury to the bile ducts.

## 2 Results

### 2.1 Engineered Biaggregate Liver Spheroids Promote Structural Hepatocyte Polarity and the Formation of Functional Bile Canaliculi

**FIGURE 1:**
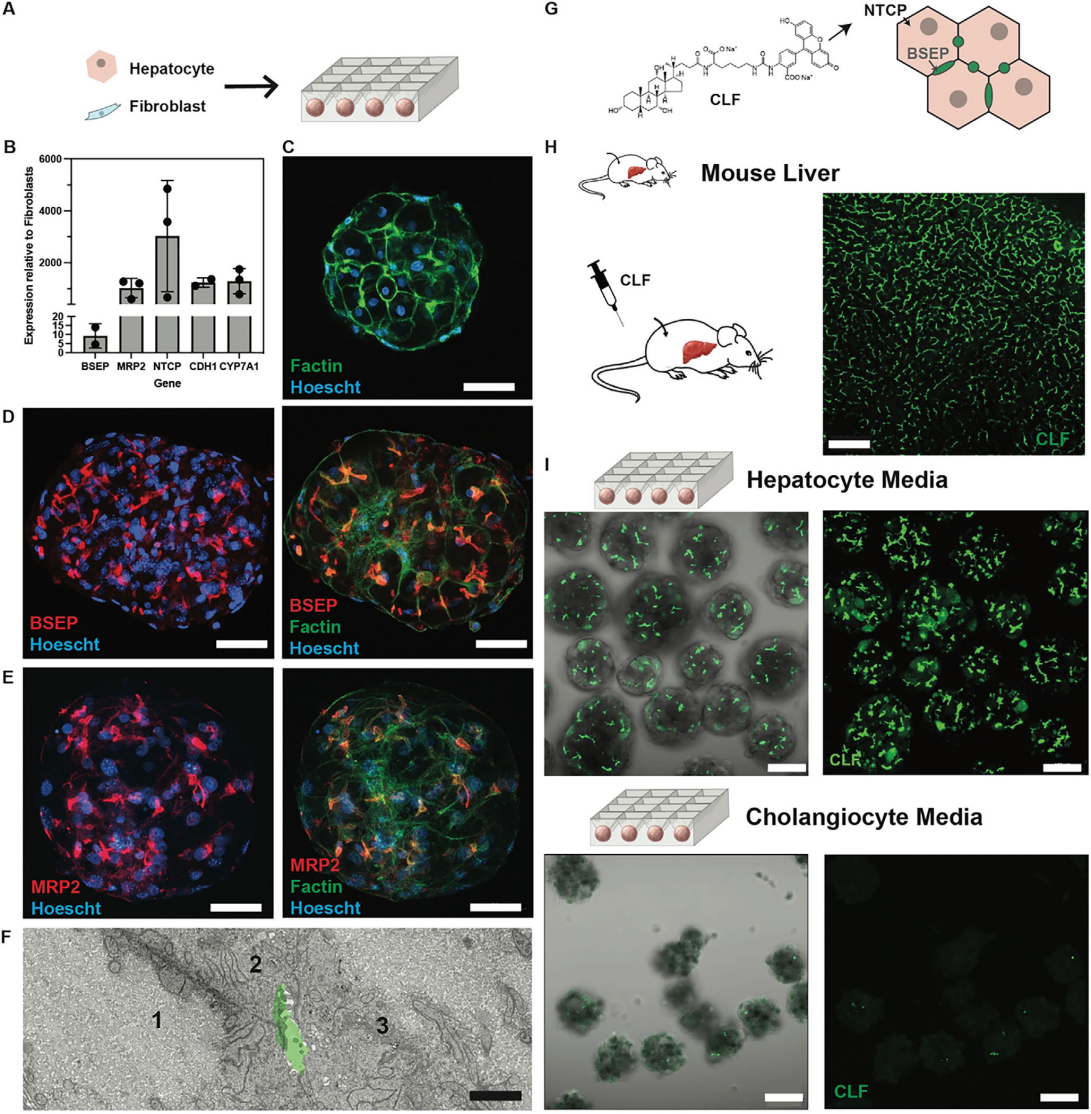
Engineered human biaggregate spheroids polarize to form bile canaliculi that secrete and transport bile acids. (A) Schematic depicting the formation of biaggregate spheroids by incubating a suspension of primary human hepatocytes and supporting mouse fibroblasts in custom V‐bottom aggrewells. (B) RNA expression of genes involved in hepatocyte polarity and bile acid synthesis, as measured by RT‐qPCR in biaggregate spheroids after 3 days in culture. Data are shown as mean ± SD with individual data points representing independent spheroid preparations (n = 3). Protein expression of F‐actin (C), BSEP (D), and MRP2 (E) in biaggregate spheroids in 3D confocal microscope images of hepatocyte spheroids after 3 days in culture, scale bars = 50 µm. (F) Representative STEM image of bile canaliculi (pseudocolored in green) in biaggregate spheroids after 3 days in culture, scale bar = 2 µm. (G) Schematic depicting the uptake and secretion of cholyl‐lysyl fluorescein (CLF) by polarized hepatocytes. H) CLF secretion in bile canaliculi of mouse liver tissue after intravenous delivery, scale bar = 100 µm. (I) CLF secretion in bile canaliculi of biaggregate spheroids after 3 days of culture in hepatocyte media (top) and cholangiocyte media (bottom), scale bars = 100 µm.

To model the tiniest branches of the biliary tree, which are comprised of the bile canaliculi formed by the apical surfaces of hepatocytes, we first sought to engineer human liver spheroids with hepatocyte polarity and functional bile transport. Building upon our lab's previously established method of liver spheroid generation [22, 23], we set out to find the media conditions that enable human hepatocytes to polarize and form bile canaliculi within biaggregate spheroids made of primary human hepatocytes and supporting fibroblasts (Figure 1a). We then characterized the expression of genes important to hepatocyte polarity and bile acid synthesis. As measured by RT‐qPCR, biaggregate liver spheroids express genes important to hepatocyte polarity at the apical (*BSEP, MRP2*), basolateral (*NTCP*), and tight junction (*CDH1*) portions of the membrane, as well as *CYP7A1*, an important gene in the first step of bile acid synthesis (Figure 1b). We show that our biaggregate spheroids polarize to form bile canaliculi through immunostaining for F‐actin (Figure 1c), which has been shown to localize at the apical surface of hepatocytes [24]. Additionally, we show that bile acid transporters are not only expressed in biaggregates at the mRNA transcript level, but also at the protein level and are localized at the apical membrane with F‐actin, highlighting the formation of structural bile canaliculi within the spheroids (Figure 1d,e). We further confirm the presence of bile canaliculi within our biaggregate spheroids using scanning transmission electron microscopy (STEM), which shows the formation of lumen structures between hepatocytes (Figure 1f).

We next established a functional readout of bile acid transport within our engineered biaggregate spheroids. To do this, we adapted an assay using cholyl‐lysyl fluorescein (CLF), a fluorescently labeled bile acid with an image‐based readout to visualize bile acid transport to the canaliculi in our engineered biaggregate spheroids (Figure 1g). This assay has previously been used to visualize bile transport to bile canaliculi in mouse liver tissue [25], in polarized rat hepatocytes within in vitro culture platforms [26], and in Canals of Hering‐like connections between murine hepatocytes and cholangiocytes [20]. We showed that CLF localizes to the bile canaliculi network in a mouse liver upon intravenous administration of CLF in a saline solution (Figure 1h). Upon performing the assay in the engineered biaggregate spheroids, we found the CLF to be transported into luminal structures that resemble the size and morphology of bile canaliculi observed in native liver tissue (Figure 1i) [27]. Next, we engineered media conditions to promote the function of these bile canaliculi in biaggregate liver spheroids. We found that media components used in standard hepatocyte medium, such as insulin‐transferrin‐selenium, fetal bovine serum, and dexamethasone, were important for canaliculi formation. In addition, we found that the inclusion of epidermal growth factor (EGF) increased bile canaliculi number, length, and width (Figure S1).

### 2.2 Hepatocyte Polarity and Bile Canalicular Function Increases in Engineered Biaggregate Spheroids Over Time

**FIGURE 2:**
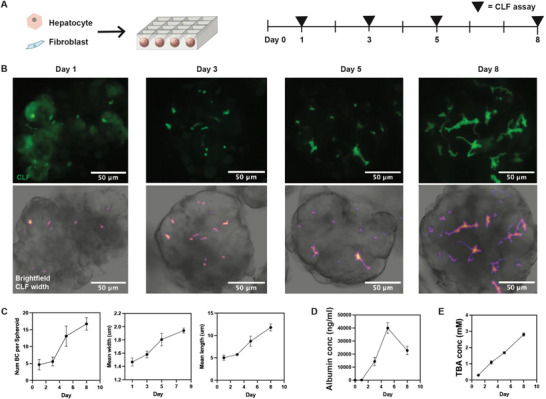
Characterization of bile acid synthesis and transport in engineered biaggregate spheroids. (A) Schematic depicting timeline of biaggregate spheroid culture and CLF assay timepoints. (B) Representative CLF (top) and width‐coded (bottom) images of biaggregate spheroids over time, scale bars = 50 µm. (C) Quantification of number (left), mean width (middle), and mean length (right) of bile canaliculi in biaggregate spheroids over time. Data are shown as mean ± SD of values quantified from n = 3 independent fields of view, each containing approximately 20 spheroids. The experiment was performed independently twice, and results from one representative experiment are shown. (D) Albumin is secreted into the media by biaggregate spheroids over time. Data are shown as mean ± SD from n = 3 independent wells from the same experiment. (E) Total bile acids are secreted into the media by biaggregate spheroids over time. Data are shown as mean ± SD from n = 3 independent wells from the same experiment.

In order to further characterize bile canalicular formation and function in our engineered biaggregate spheroids, we cultured the biaggregate spheroids over a period of eight days and performed the CLF assay longitudinally (Figure 2a,b). We developed an image analysis pipeline to measure the number, length, and width of bile canaliculi based on CLF signal in biaggregate spheroids (Figure 2b,c). Quantification of several spheroids revealed that bile canaliculi increased in number, length, and width over time in culture (Figure 2c). These measurements fell within the range of established values for human bile canaliculi, reported to range between 1 and 3 µm in width [27, 28]. We also confirmed that our spheroids produced albumin, an important indicator of hepatocyte maturity and function, at levels within the range reported for human hepatocytes in vitro (Figure 2d) [29, 30]. We also observed the steady increase in total bile acid (TBA) concentration in the media (Figure 2e), indicating that biaggregate spheroids produce and secrete bile acids into the culture media.

### 2.3 Engineered Adult Hepatobiliary Organoids (aHBOs) Recapitulate Physiological Bile Transport Function

**FIGURE 3:**
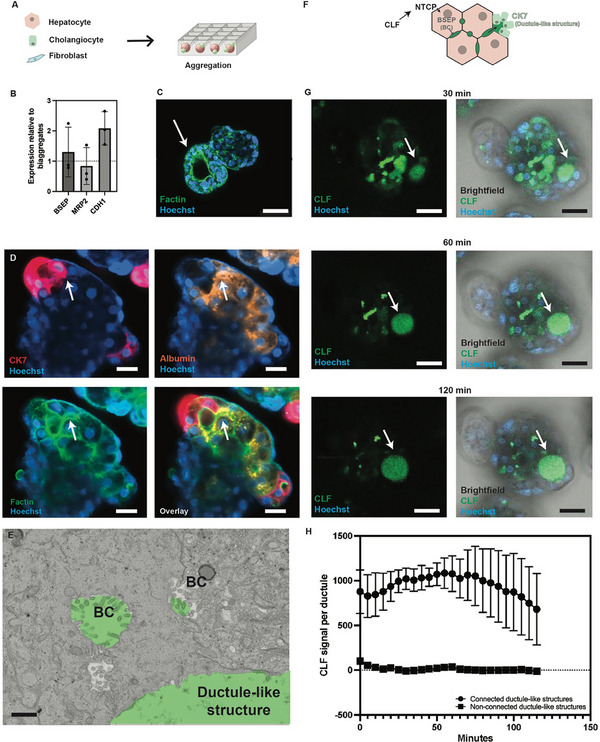
Engineered human adult hepatobiliary organoids (aHBOs) contain hepatobiliary junctions that transport bile from bile canaliculi to intrahepatic ductule‐like structures. (A) Schematic depicting the formation of aHBOs with hepatobiliary junctions, by incubating a suspension of primary human hepatocytes, human intrahepatic cholangiocytes, and supporting mouse fibroblasts in custom V‐bottom aggrewells. (B) RNA expression of genes involved in hepatocyte polarity, as measured by RT‐qPCR in aHBOs after 3 days in culture. Data are shown as mean ± SD with individual data points representing independent spheroid preparations (n = 3). (C) Protein expression and localization of F‐actin (green) in a representative confocal microscope image of aHBO with ductule‐like structure (arrow) after 3 days in culture, scale bar = 50 µm. (D) Protein expression and localization of CK7 (red), albumin (orange), and F‐actin (green), demonstrating hepatobiliary connection in a single aHBO after 3 days in culture. White arrows point to F‐actin‐lined hepatobiliary connection between CK7+ ductule‐like structure and albumin+ hepatocytes, scale bars = 25 µm. For full images collected from a 3D z‐stack, see Video S2. (E) Representative STEM images of bile canaliculi (BC) and a ductule‐like structure (pseudocolored green) in aHBOs after 3 days in culture, scale bar = 1 µm. (F) Schematic depicting the transport of CLF from hepatocyte bile canaliculi to ductule‐like structures. (G) Still images captured from time‐lapse of bile acid transport in aHBOs at 30 (top), 60 (middle), and 120 (bottom) minutes after incubation with CLF (green), scale bars = 50 µm. White arrows indicate ductule‐like structures filling with CLF over time. For full timelapse, see Video S1. (H) Quantification of timelapse images of CLF transport in aHBOs in connected ductule‐like structures (circle) and non‐connected ductule‐like structures (square), beginning after incubation in CLF and subsequent wash steps. Data are shown as mean ± SD from n = 3 connected and n = 3 non‐connected ductule‐like structures identified from spheroids in the same field of view.

After constructing a model system that structurally and functionally recapitulates the smallest elements of the biliary system, we set out to add the next element of the hepatobiliary junction: intrahepatic ductules that functionally connect to bile canaliculi. To do this, we isolated intrahepatic cholangiocytes from adult human liver biopsy tissue, establishing a reliable source of human intrahepatic cholangiocytes. We cultured and expanded these cells as organoids in 3D Matrigel culture (Figure S2), as previously described [16, 17]. Next, we sought to identify the co‐culture conditions necessary to not only enable the mutual survival of both hepatocytes and cholangiocytes but also to facilitate the structural and functional connection between these two cell types, thus creating the minimal functional unit of the hepatobiliary system. To this end, we modified our spheroid aggregation system to generate triaggregate spheroids, consisting of primary human hepatocytes, intrahepatic cholangiocytes, and supporting mouse fibroblasts (Figure 3a). We tested a variety of cell ratios and media conditions, and empirically determined that a mixed co‐culture media and a 2:2:1 ratio of hepatocytes, fibroblasts, and cholangiocytes enabled the formation of adult hepatobiliary organoids (aHBOs). We found, through RT‐qPCR, that aHBOs expressed hepatocyte polarity genes at a similar level to biaggregates (Figure 3b). Staining with F‐actin, CK7, and albumin revealed the presence of both cholangiocytes and hepatocytes within the same aHBO, indicating the survival and polarity of both cell types within our engineered culture (Figure 3c,d). Our immunostaining revealed the presence of ductule‐like structures expressing cholangiocyte marker, cytokeratin‐7 (CK7), as well as the presence of F‐actin‐lined hepatobiliary connections between CK7+ cholangiocytes and albumin+ hepatocytes (Figure 3d and Video S1). Additionally, hepatocytes and cholangiocytes maintain polarity in aHBOs, as shown by immunostaining of BSEP at bile canaliculi and E‐cadherin in ductule‐like structures (Figure S4). STEM imaging also confirmed the presence of bile canaliculi and ductule‐like structures within the same spheroid (Figure 3e).

After confirmation that the structural elements of the hepatobiliary junction were present, we sought to characterize the bile transport function within our engineered spheroids. To do this, we used the CLF assay to visualize the connection between bile canaliculi and ductule‐like structures (Figure 3f). Time‐lapse imaging of CLF transport in aHBOs demonstrated the initial accumulation of CLF first in bile canaliculi at 30 min after incubation (Figure 3g, top & Video S2). At 60 min, the CLF drained from bile canaliculi to connected ductule‐like structures, with the CLF signal within the ductule‐like structures increasing steadily (Figure 3g, middle and Video S2). By 120 min, the CLF signal in the bile canaliculi had dramatically decreased with accumulation of signal in connected ductule‐like structures (Figure 3g, bottom & Video S2). This accumulation of CLF signal did not occur in all ductule‐like structures, implying that the CLF collection was only able to occur in ductule‐like structures functionally connected to bile canaliculi from which the CLF drained. Non‐connected ductule‐like structures failed to fluoresce with CLF signal throughout the entire 120‐min duration of the experiment (Figure 3h).

### 2.4 Characterizing Hepatobiliary Junction Dynamics in aHBOs Over Time

**FIGURE 4:**
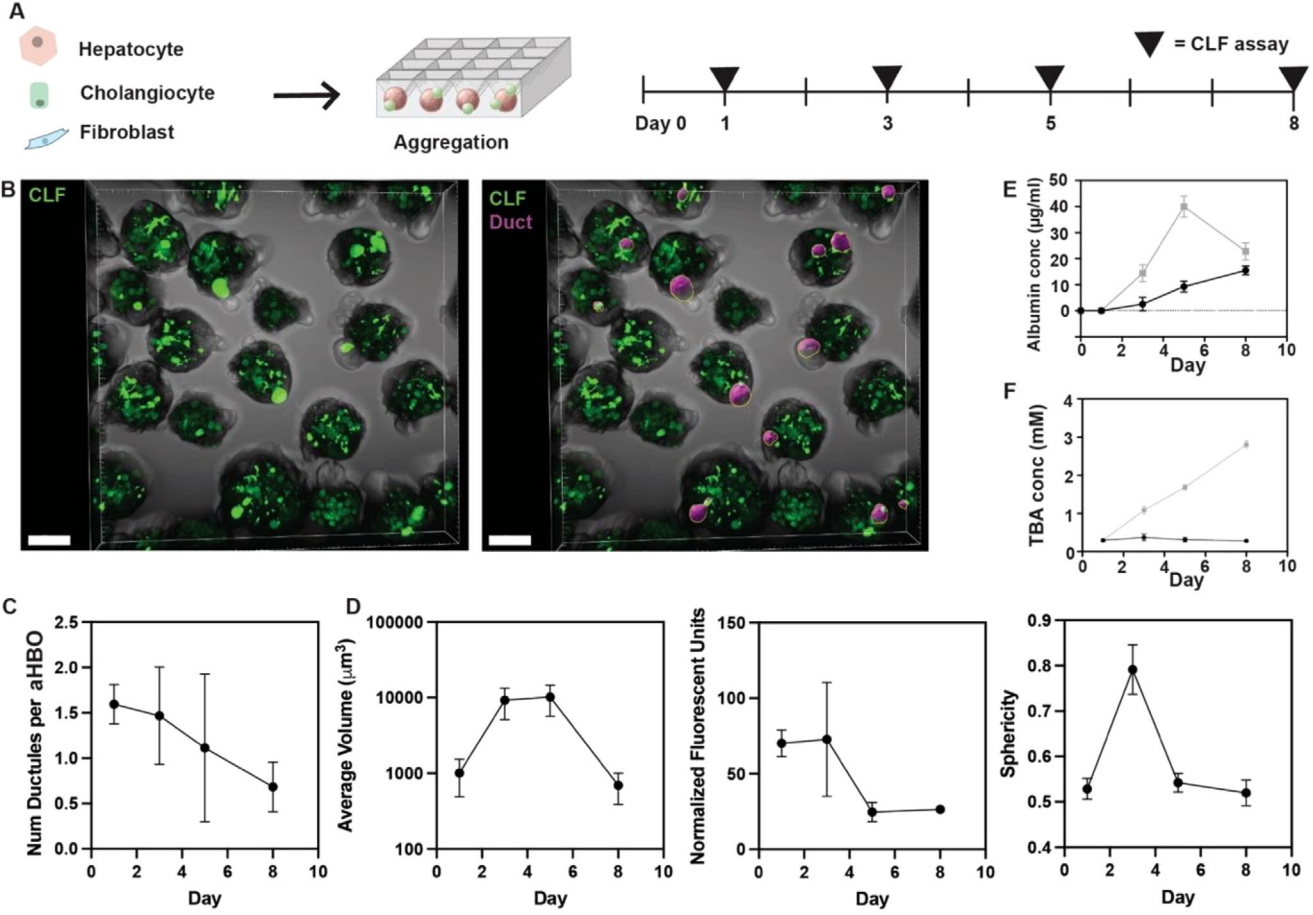
Functional characterization of intrahepatic ductule‐like structures in engineered aHBOs. (A) Schematic depicting timeline of aHBO culture and CLF assay timepoints. (B) Representative images of CLF transport from bile canaliculi to bile ductule‐like structures in aHBOs (left) and segmentation of CLF signal in bile ductule‐like structures (right, purple), scale bar = 100 µm. (C) Quantification of the number of CLF‐positive ductule‐like structures per aHBO over time. Data are shown as mean ± SD of values quantified from n = 3 independent fields of view, each containing approximately 20 spheroids. The experiment was performed independently three times, and results from one representative experiment are shown. (D) Quantification of volume (left), fluorescence intensity (middle), and sphericity (right) of CLF‐positive bile ductule‐like structures in aHBOs over time. Data are shown as mean ± SD of values quantified from n = 3 independent fields of view, each containing approximately 20 spheroids. The experiment was performed independently three times, and results from one representative experiment are shown. (E) Albumin secreted into media by aHBOs (black) and biaggregate spheroids (gray) over time. For the purposes of making a direct comparison, biaggregate data shown in gray is overlaid with data also shown in Figure 2d. Data are shown as mean ± SD from n = 3 independent wells from the same experiment. (F) Total bile acids secreted into media by aHBOs (black) and biaggregate (gray) spheroids over time. For the purposes of making a direct comparison, the biaggregate data shown in grey is overlayed with data also shown in Figure 2e. Data are shown as mean ± SD from n = 3 independent wells from the same experiment.

In our newly developed system, we sought to characterize hepatobiliary junction formation and function over a longitudinal period. To do this, we seeded aHBOs and cultured them for varying lengths of time before initiating the CLF assay, with 8 days being the longest continuous culture time (Figure 4a). We then implemented an image analysis pipeline that leveraged an AI‐guided image segmentation tool to identify CLF‐positive ductule‐like structures in a 3D reconstruction of images collected during the CLF assay (Figure 4b). From this image analysis, we were able to determine the number of CLF‐positive ductule‐like structures in each image, normalized by the number of aHBOs in the image (Figure 4c).

To determine how much culture time was necessary for these connections to form, we measured volume and fluorescence intensity of CLF‐positive ductule‐like structures within aHBOs cultured for different periods of time, ranging from 1 day to 8 days. While the volume of these ductule‐like structures peaked in size between days 3 and 5 in culture (Figure 4d, left), the fluorescence intensity decreased but still showed a positive CLF signal out to 8 days in culture (Figure 4d, middle), indicating that aHBOs contained connected hepatobiliary junctions even after a prolonged continuous culture period. We also quantified sphericity as a readout of ductule‐like structure morphology over time. The 3‐day‐old aHBOs contained ductule‐like structures with high sphericity, and while the ductule‐like structures in 5‐day‐old and 8‐day‐old aHBOs decreased in sphericity, they still collected CLF, as indicated by the positive fluorescence intensity in spheroids at these culture times (Figure 4d, right). Taken together, these results show that in these culture conditions, hepatobiliary junction function peaks in aHBOs around 3 days in culture, but can be sustained up to 8 days in culture.

Lastly, we measured albumin and bile acid synthesis functions in aHBOs by performing an albumin ELISA and a total bile acid (TBA) assay on media collected from these cultures at various timepoints. We show that aHBOs exhibit sustained albumin secretion, consistent with reported values for primary human hepatocytes in spheroid cultures (Figure 4e) [30]. However, we see that TBA concentration in media collected from aHBOs is much less than that collected from biaggregates (Figure 4f). This finding suggests that bile acids may be accumulating in the ductule‐like structures of the aHBOs, an indication of physiological intrahepatic biliary function. These results demonstrate directional bile transport from hepatocytes to a connected ductular lumen within the aHBO system, a feature consistent with intrahepatic bile duct function.

### 2.5 Hypoxia‐Reoxygenation Disrupts Bile Flow Within aHBOs and Reveals Differential Cellular Responses to Biliary Hypoxic Stress

We next aimed to determine how hepatobiliary function in aHBOs responds to changes in oxygen availability. In vivo, bile duct ischemic injury following liver transplantation manifests in several clinically distinct forms. Anastomotic biliary strictures are thought to arise from localized hypoxia due to disruption of the biliary blood supply during surgical transection. In contrast, non‐anastomotic strictures are less well understood but are commonly associated with ischemia–reperfusion injury and delayed graft function [31]. More severe ischemic injury, such as that caused by hepatic artery thrombosis, can lead to ischemic cholangiopathy characterized by biliary necrosis, intrahepatic abscess formation, and graft loss. Together, these conditions highlight the vulnerability of the biliary epithelium to oxygen deprivation. As such, developing in vitro models that capture important aspects of these disease phenotypes, including hepatobiliary architecture and bile flow, may potentially contribute to the treatment landscape.

**FIGURE 5:**
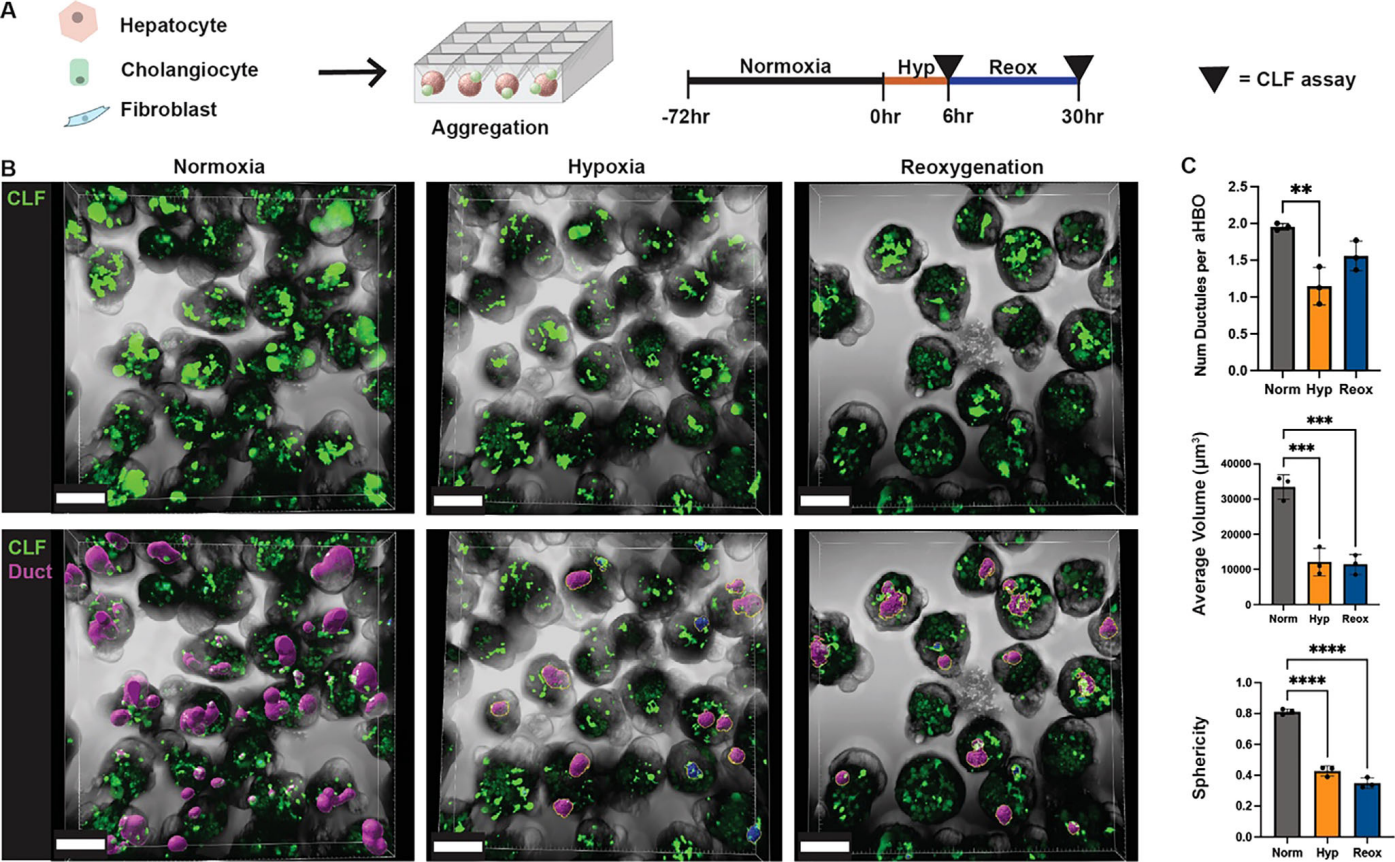
Hypoxia‐reoxygenation disrupts bile flow within engineered aHBOs. (A) Schematic depicting the timeline of hypoxia‐reoxygenation conditioning of aHBOs and CLF assay timepoints. (B) Representative images of CLF transport from bile canaliculi to bile ductule‐like structures from independent wells of aHBOs in normoxic (top left), hypoxic (top middle), and reoxygenated (top right) conditions, and segmentation of CLF signal in bile ductule‐like structures (bottom, purple), scale bar = 100 µm. Representative images were acquired from separate fields in independent wells. (C) Quantification of the number (top), volume (middle), and sphericity (bottom) of CLF‐positive ductule‐like structures in each condition. Data are shown as mean ± SD of values quantified from n = 3 independent fields of view, each containing approximately 20 spheroids. The experiment was performed independently twice, and results from one representative experiment are shown. Statistical significance was assessed by one‐way ANOVA with post hoc multiple‐comparisons testing; significance was observed only between the groups indicated by brackets and asterisks.

While transport timelines for donor livers can vary widely from patient to patient, the average ischemia time for a donor liver is around 6 h, during which time the liver is cut off from blood supply before being reconnected to the organ recipient's systemic circulation [32]. To model kinetics of the typical ischemia‐reperfusion process that occurs during liver transplantation, after 3 days in normoxic (21% oxygen tension) culture, we subjected aHBOs to 6 h of hypoxia (2% oxygen tension) and then reoxygenated the spheroids for 24 h. We conducted the CLF assay in normoxia, after 6 h of hypoxia, and after 24 h of reoxygenation (Figure 5a). We then detected the CLF‐positive ductule‐like structures at these timepoints (Figure 5b). Under hypoxia and hypoxia‐reoxygenation, significantly fewer CLF‐positive ductule‐like structures were detected than in normoxia (Figure 5c, top). Notably, under hypoxic conditions, CLF‐positive ductule‐like structures have a significantly decreased volume (Figure 5c, middle) and sphericity (Figure 5c, bottom), and the changes to these features seem to persist upon reoxygenation. Taken together, the presence of smaller, less spherical, and more irregularly shaped ductule‐like structures under hypoxia–reoxygenation suggests that this conditioning may contribute to a volumetric collapse of the ductule‐like structures. Biliary collapse or stricturing is a key phenotype observed in ischemia‐reperfusion injury [33], and the observed changes in aHBO morphology may reflect aspects of this phenotype, motivating future studies to further define the extent to which this model captures ischemic cholangiopathy‐associated features. While hypoxia‐reoxygenation is used here to perturb hepatobiliary function in a manner relevant to liver transplantation, this conditioning is not intended to fully recapitulate any single post‐transplant biliary disease. Rather, our setup enabled controlled interrogation of hypoxia‐induced changes in both bile transport and structural integrity of the hepatobiliary junction.

### 2.6 Reversible Bile Canalicular Transport Decline Under Hypoxia is Followed by Loss of Cholangiocyte Viability Under Reoxygenation of aHBOs

**FIGURE 6:**
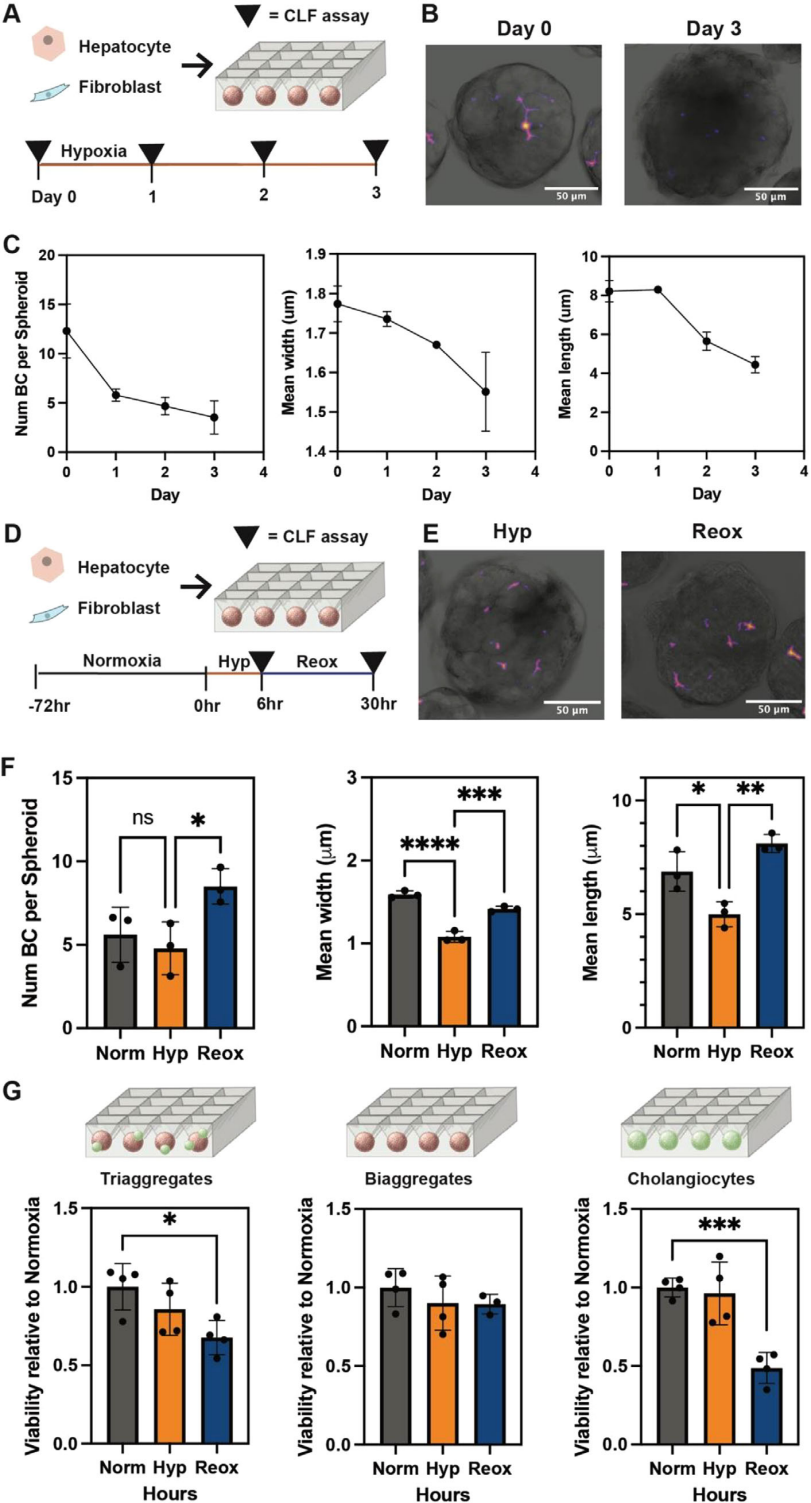
Hypoxia disrupts bile canalicular transport while reoxygenation triggers cholangiocyte‐specific cell death in engineered spheroids (A). Biaggregate spheroids were cultured in hypoxia for 3 days, with CLF assay performed on each day. (B) Representative images of bile canaliculi in biaggregate spheroids in normoxia (left) and after 3 days of hypoxia (right), scale bars = 50 µm. (C) Quantification of bile canaliculi number (left), width (middle), and length (right) over time in hypoxia. Data are shown as mean ± SD of values quantified from n = 3 independent fields of view, each containing approximately 20 spheroids. The experiment was performed independently twice, and results from one representative experiment are shown. (D) Biaggregate spheroids were cultured in hypoxia for 6 h and then reoxygenated for 24 h. (E) Representative images of bile canaliculi in biaggregate spheroids after 6 h of hypoxia (left) and 24 h of reoxygenation (right), scale bars = 50 µm. (F) Quantification of bile canaliculi number (left), width (middle), and length (right) in hypoxia‐reoxygenation conditioning. Data are shown as mean ± SD of values quantified from n = 3 independent fields of view, each containing approximately 20 spheroids. Statistical significance was assessed by one‐way ANOVA with post hoc multiple‐comparisons testing; significance was observed only between the groups indicated by brackets and asterisks. (G) Viability of aHBOs (left), biaggregate spheroids (middle), and cholangiocyte spheroids (right) during hypoxia‐reoxygenation conditioning. Data are shown as mean ± SD of values quantified from n = 3 independent fields of view, each containing approximately 20 spheroids. Statistical significance was assessed by one‐way ANOVA with post hoc multiple‐comparisons testing; significance was observed only between the groups indicated by brackets and asterisks.

Upon observation of biliary dysfunction in aHBOs under hypoxia‐reoxygenation conditioning, we sought to conduct this conditioning on the individual cell types in aHBOs in order to understand the processes potentially contributing to this phenotype. First, we aimed to elucidate the effects of hypoxia on CLF transport by hepatocytes into bile canaliculi. To do this, we conditioned our biaggregate spheroids in hypoxia over the course of 3 days and conducted the CLF assay each day (Figure 6a). CLF signal in bile canaliculi decreased in number, width, and length over the course of three days (Figure 6b,c), demonstrating that hepatocyte polarity and bile transport function decrease under hypoxic conditions. To determine whether hepatocyte polarity was recoverable upon reoxygenation, we exposed biaggregate spheroids to the same hypoxia‐reoxygenation conditioning regimen that induced phenotypic changes within ductule‐like structures in aHBOs and conducted the CLF assay in the biaggregate spheroids (Figure 6d). Under hypoxia, the number of bile canaliculi stayed the same, but the width and length decreased, indicating subtle yet detectable decreases in bile canalicular function (Figure 6e, f). This decrease was recovered upon reoxygenation (Figure 6f), indicating that canalicular transport of bile can be recovered after 6 h of hypoxia within this system.

**FIGURE 7:**
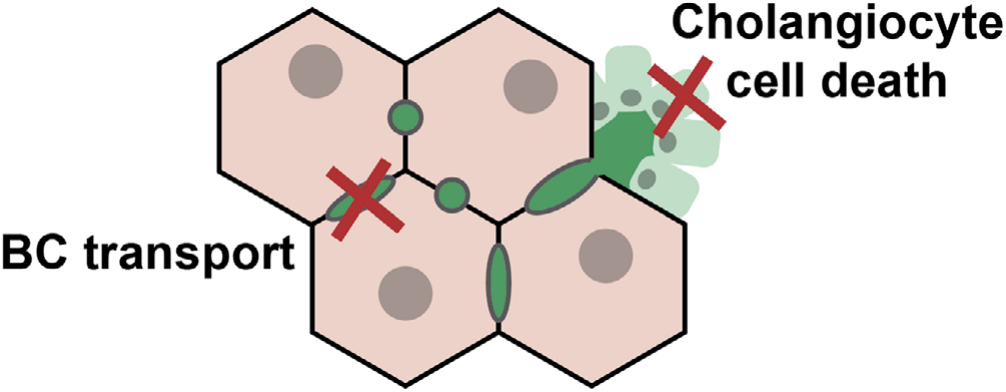
Two potential processes contributing to biliary dysfunction in post‐transplant ischemia‐reperfusion injury.

We then wanted to test the effect of hypoxia‐reoxygenation on cell viability within aHBOs. We observed a slight decline in total cell viability in aHBOs under hypoxia, and this decline was further exaggerated under reoxygenation (Figure 6g, left). In our biaggregate spheroids, cell viability was unchanged in hypoxia and reoxygenation (Figure 6g, middle), while spheroids consisting of only cholangiocytes significantly decreased in viability after hypoxia‐reoxygenation (Figure 6g, right). This pattern suggests that the observed decrease in aHBO cell viability can be attributed to the death of cholangiocytes. Taken together, these findings are consistent with a pair of processes that contribute to biliary dysfunction following changes in aHBO oxygenation: The 1) reversible decrease in hepatocyte bile canalicular function under hypoxia, coupled with 2) increased cholangiocyte‐specific death following reoxygenation, leads to a volumetric collapse of ductule‐like structures in our hepatobiliary units (Figure 7). Through this work, we establish a culture model of aHBOs that demonstrate bile flow within a healthy hepatobiliary unit, and that may also be used to study the mechanisms of clinically‐relevant hepatobiliary diseases.

## 3 Discussion

In this paper, we engineered a 3D in vitro model of the human hepatobiliary junction in health and disease by leveraging a combination of spheroid aggregation and intrahepatic cholangiocyte organoid technology. Specifically, we optimized the culture conditions in which primary human hepatocytes establish polarity and transport bile acids into bile canaliculi within a spheroid context. To visualize and quantify this feature, we developed an image‐based assay for high‐throughput quantification of bile transport into canaliculi. After establishing a primary human intrahepatic cholangiocyte organoid line, we incorporated these cells into our spheroid platform to engineer aHBOs, which contain a functional hepatobiliary junction between hepatocytes and cholangiocytes. We next adapted our image‐based assay to characterize and quantify junction dynamics in aHBOs over time. We then observed bile transport in aHBOs under conditions of hypoxia and reoxygenation, which models one of the phenotypic changes seen in post‐transplant ischemia‐reperfusion injury. Recent studies have demonstrated that intrahepatic cholangiocytes undergo intrinsic apoptosis when stressed by hypoxia, followed by extrinsic apoptosis and necroptosis upon reoxygenation, often coupled with upregulation of epithelial to mesenchymal transition (EMT)‐associated markers and disintegration of the actin skeleton [34, 35]. While these findings elucidate the cholangiocyte‐specific mechanisms of ischemic injury in a monoculture context, the causes and consequences of biliary dysfunction in ischemic cholangiopathy are intricately linked to bile flow [36], [37], which requires a model system that incorporates a functional hepatobiliary junction to study. In this work, we develop such a model system and build upon recent findings by studying the effect of hypoxia‐reoxygenation conditioning in the context of bile transport in a 3D multicellular environment. It is important to note that the hypoxia‐reoxygenation paradigm we have employed does not model the full clinical complexity of post‐transplant ischemic cholangiopathy. Instead, this approach serves as a controlled perturbation to examine how hepatocytes and cholangiocytes within a functional hepatobiliary junction respond differentially to oxygen deprivation and reoxygenation. Recapitulation of hepatobiliary connections enables investigation of cholangiocyte responses to hypoxia‐reoxygenation within a physiologically relevant biliary architecture, alongside concurrent assessment of hepatocyte canalicular function. As such, aHBOs serve as a platform for generating hypotheses regarding the potential processes that contribute to biliary dysfunction in the setting of liver transplantation. These hypotheses include changes in intraluminal pressure caused by the reversible reduction in canalicular flow in hypoxia, as well as compromises in barrier function triggered by cholangiocyte apoptosis in reoxygenation. These hypotheses provide a basis for several future studies.

One limitation of our system is the use of a non‐physiological ratio of cholangiocytes to hepatocytes; while our system incorporates a 2:1 ratio of hepatocytes to cholangiocytes, the native liver has much fewer cholangiocytes, with a ratio closer to 16:1 [38]. We justify this design choice to maximize the number of hepatobiliary junctions generated by our platform, such that perturbations to this junction can be observed and averaged over many replicates. In fact, the high‐throughput nature of our engineered model system opens the door to a plethora of future screen‐based studies. Because the CLF assay serves as a high‐throughput, image‐based functional readout for connected vs. unconnected ductule‐like structures, functional genomics screens can be used to elucidate the genetic basis of hierarchical bile transport. Additionally, aHBOs can potentially be used in high‐throughput compound screening to develop therapeutics that prevent or reverse biliary dysfunction in patients with ischemic cholangiopathy or, more broadly, patients with a range of cholestatic diseases. Beyond transplantation‐associated biliary hypoxia, this platform may be particularly well suited for modeling drug‐induced cholestasis or genetic disorders affecting canalicular transport. These applications will be explored in future studies.

While the use of murine fibroblasts in our spheroid aggregation renders our system chimeric, the cell types that make up the hepatobiliary junction are human‐derived. Our lab has previously shown that fibroblasts are essential for the formation of biaggregates and maintenance of hepatocyte function in culture, through providing key paracrine and juxtacrine signals [39, 40]. Given prior reports that fibroblasts promote cholangiocyte proliferation in liver organoid models [41], we optimized the aHBO cell ratio to balance support for hepatocyte function with controlled cholangiocyte expansion for the formation of ductule‐like structures. We strategically chose a murine fibroblast source so that using human primers in our bulk gene expression analyses (RT‐qPCR and bulk RNA sequencing) would reveal gene expression of only the human hepatobiliary cell types within this system, without noise from the murine fibroblasts. Recent studies have pointed to the role of fibroblasts in biliary fibrosis by generating an in vitro murine and human liver models in which tuning the ratio of portal fibroblasts induces biliary fibrosis independently of an immune compartment [41, 42]. Specifically, a recent publication by Yuan et al. introduced a similar modular strategy for assembling human hepatocytes, cholangiocytes, and fibroblasts in vitro, using an approach not unlike the one we describe [42]. Their system optimized media conditions to promote hepatocyte proliferation in the coculture model, but only upon differentiation media conditions did the hepatocytes form mature bile canalicular structures. In contrast, our aHBO platform incorporates mature adult human hepatocytes directly, without inducing proliferation, enabling stable bile canalicular formation under coculture conditions. Furthermore, our work builds upon these approaches by integrating a high‐throughput functional imaging pipeline using the CLF assay to quantitatively assess bile canalicular transport and hepatobiliary connectivity. In addition, Yuan et al. Highlight the critical role of fibroblasts and how tuning their ratio can cause profound effects on the viability and phenotype of both liver parenchymal cell types. This study aligns with our work, in which we employ a fibroblast ratio that simultaneously supports cholangiocyte expansion while preserving hepatocyte viability and canalicular function.

Besides fibroblasts, immune cells of the liver microenvironment also contribute to several liver and bile duct diseases. One recent advance in intestinal and liver organoid technology is the integration of macrophages [43, 44]. While these models have focused on viral infection, similar techniques to incorporate an immune component into aHBOs could facilitate future studies in modeling autoimmune cholestatic diseases in which immune cells play a key role, such as primary biliary cholangitis.

Our lab has previously developed liver‐on‐chip models, which incorporate vascular [45] and bile duct [46] function. Future studies could involve incorporating aHBOs into a microfluidic device‐based system that recapitulates the entire hepatic sinusoid in a 3D context, in order to model the synthesis, modification, and recirculation of bile from blood to bile duct, which is a process that is crucial in health and disease [47]. Recent engineered models have been developed to incorporate systemic vasculature [45] and bile ducts [48]. It follows that a complex in vitro system that incorporates relevant human liver cells and facilitates access to the lumen of blood vessels and bile ducts could enable us to study these processes in further detail.

In addition to modeling human tissues for in vitro studies, the field of regenerative medicine has been advancing toward engineering liver tissues with direct therapeutic utility. We and others have previously developed implantable engineered liver tissues that expand and perform important hepatocyte functions in vivo [23, 49]. While these tissues demonstrate hepatocyte polarity in vivo, they do not form a contiguous bile canalicular network that functionally drains to a cholangiocyte‐lined bile duct, which is a crucial function for the future of engineered liver tissues that can serve as a therapy for patients with cholestatic and bile duct diseases. Incorporating aHBOs into an implantable engineered liver tissue could serve as a promising next step toward developing a fully‐functional liver tissue replacement.

Overall, we describe the development of adult human hepatobiliary organoids that capture key features of physiological and pathological intrahepatic bile duct function.

## 4 Methods

### 4.1 Human Intrahepatic Cholangiocyte Organoid (ICO) Initiation and Culture

For initiation of organoid cultures, a fresh donor liver biopsy from a 54‐year‐old male was collected and stored in cold University of Wisconsin solution. The patient gave informed consent for the use of material for research purposes. This study was reviewed by the Institutional Review Board (IRB) at MIT and determined to be exempt from review under 45 CFR 46.104(d)(4) (Exempt Category 4) (IRB determination ID: E‐4709).

A human ICO line was initiated and cultured using an established protocol [14]. In brief, donor liver tissue was minced using a scalpel, then washed twice via centrifugation in William's E medium supplemented with 50 ng/mL EGF and 10 µM Y27632. The cells were then seeded in basement membrane extract (R&D Systems) in multi‐well plates. Organoid initiation medium, comprised of ICO expansion media (Table S1) supplemented with 25 ng/mL recombinant human Noggin (Peprotech 120‐10C), 30% Wnt3a‐conditioned media (made in‐house), and 10 µM Y27632 (SelleckChem, S1049), was then added to the culture. After three days of culture in the initiation medium, the ICO expansion media was added to the cells. ICOs were grown and passaged in ICO expansion medium as previously described [14, 16] for 3–6 passages before inclusion in aHBOs. For inclusion in aHBOs, ICO cultures were treated with 5U/ml dispase II (Stemcell Technologies, 07913) until the basement membrane extract was dissolved, then TrypLE Express Enzyme (Gibco, 12604021) until a single‐cell suspension of cholangiocytes was generated.

### 4.2 Cell Culture

Primary cryopreserved human hepatocytes were sourced from a commercial vendor (Thermo Fisher, Lot #4129) and maintained in Hepatocyte Media (see Table S1). 3T3‐J2 murine fibroblasts were a kind gift provided by Howard Green (Harvard Medical School) and were cultured in DMEM with 4.5 g/L glucose (Corning, 10‐017‐CV), 10% fetal bovine serum, and 1% (v/v) penicillin‐streptomycin. Upon confluence, fibroblasts were passaged by treatment with 0.25% Trypsin (Corning, 25‐050‐CI).

### 4.3 Spheroid and aHBO Formation

AggreWell plates containing 400 µm pyramidal microwells were fabricated in‐house and prepared as described previously [22]. To generate biaggregate spheroids, cryopreserved human hepatocytes were thawed and mixed with fibroblasts in a 1:1 ratio of hepatocytes to fibroblasts. The two‐cell mixture was then plated in AggreWell plates in hepatocyte media (Table S1) over the indicated period of time. Cell numbers were optimized for a 12‐well AggreWell plate; each well was seeded with 0.25 millionm hepatocytes and 0.25 millionm fibroblasts.

Prior to establishing an HBOs, bile canaliculi formation was assessed across hepatocyte spheroids generated from 17 donors obtained from 3 independent vendors, confirming consistent CLF phenotypes across donors (Figure S5). For the experiments described in this paper, a single, well‐characterized donor (Thermo Fisher, Lot #4129) was selected to eliminate donor‐to‐donor variability across our experiments and to enable controlled comparisons across experimental conditions. This approach is consistent with prior studies utilizing primary hepatocytes, where reducing biological variability facilitates interpretation of complex multicellular systems, but our findings are generalizable to multiple human hepatocyte donors.

To generate aHBOs, cryopreserved human hepatocytes were thawed and mixed with fibroblasts and single‐cellularized ICOs, in a 2:2:1 ratio of hepatocytes to fibroblasts to ICOs. The three‐cell mixture was then plated in AggreWells in a coculture media comprised of 50% Hepatocyte Media and 50% Cholangiocyte Media (Table S1). In a 12‐well AggreWell plate, each well was seeded with 0.25 millionm hepatocytes, 0.25 millionm fibroblasts, and 0.125 millionm single‐cellularized ICOs. To create a single‐cellularized suspension of ICOs, ICO cultures were treated with 5U/ml dispase II (Stemcell Technologies, 07913) until the basement membrane extract was dissolved, then TrypLE Express Enzyme (Gibco, 12604021) until a single‐cell suspension of cholangiocytes was generated.

### 4.4 Hypoxia and Reoxygenation Stimulation in aHBOs

After three days of aggregation in coculture medium (Table S1), aHBOs were transferred to an incubator with hypoxic 1–21% oxygen control (Thermo Fisher, Model 3140), with gas settings at 2% O2, 5% CO2 for 6 h. aHBOs were then cultured in normoxia (21% O2, 5% CO2) for a reoxygenation time of 24 h.

### 4.5 RNA Isolation and RT‐qPCR

To measure gene expression via RT‐qPCR, the media was first removed, then the sample was lysed and homogenized in TRIzol reagent (Invitrogen, 15596026). RNA was isolated via chloroform extraction and further purified with the RNeasy MinElute Cleanup Kit (QIAGEN, 74204). cDNA was synthesized using RevertAid First Strand cDNA Synthesis Kit (Thermo Fisher, K1622), according to the manufacturer's instructions. RT‐qPCR was performed using PowerUp SYBR Green Master Mix (Thermo Fisher, A25742) in a Bio‐Rad CFX96 Real‐Time PCR Detection System, according to the manufacturer's instructions. The primer sequences used to detect mRNA levels are listed in Table S2. Relative mRNA quantification was calculated with the Delta‐delta Ct method, using *HMBS* as a housekeeping gene.

### 4.6 Immunostaining

For immunostaining, spheroids were fixed in 4% paraformaldehyde in PBS for 30 min–1 h. Spheroids were then blocked and permeabilized in 3% bovine serum albumin (BSA) with 0.05% Triton X‐100 for 3 h. Spheroids were then incubated in primary antibody diluted in 3% BSA (according to the vendor's recommendation) at 4C overnight. Spheroids were washed 5 times for 15 min each in PBS, then incubated in secondary antibodies (1:500 dilution) and Hoechst 33342 (1:2000) in 3% BSA at 4C overnight. Spheroids were washed 5 times for 15 min each in PBS before being mounted on a coverslip with ProLong Diamond Antifade Mountant (Invitrogen, P36961) and imaged.

### 4.7 Confocal Imaging

Images were obtained on a Zeiss LSM 900 confocal microscope with Airyscan super‐resolution detector (Carl Zeiss, Jena, Germany), and processed using ZEN software (Carl Zeiss). Immunostained spheroids were immobilized on a coverslip and imaged using a Plan‐Apochromat 20x/0.8 objective (Carl Zeiss, 440640‐9903‐000).

### 4.8 In Vitro CLF Assay

Spheroids were collected from AggreWell plates, gently pelleted at 100 g, and incubated in media with 5 µM cholyl‐lysyl fluorescein at 37C for 30 min. Spheroids were briefly washed three times in Advanced DMEM/F12 with 1% penicillin‐streptomycin, then plated on a coverslip and imaged in suspension. The same objective, laser intensity, and z‐stack interval were used for all samples. For each condition, at least three representative fields of view were captured and analyzed.

### 4.9 In Vivo CLF Assay

All animal experiments were conducted following Institutional Animal Care and Use Committee‐approved protocols (approval number: 0721‐060‐24). BALB/c mice (Taconic) were anesthetized using isoflurane. 100 ul of a solution of 1 mg/kg CLF in sterile saline was injected intravenously and after approximately 3 min, the mice were sacrificed, and the liver was harvested. The explanted liver was sectioned into 100–200 µm thick sections with a vibratome (Leica, VT1000S), and imaged on a coverslip using a Plan‐Apochromat 20x/0.8 objective (Carl Zeiss, 440640‐9903‐000).

### 4.10 Scanning Transmission Electron Microscopy (STEM) Imaging

Spheroids and aHBOs were initially fixed with 2.5% glutaraldehyde, 2% paraformaldehyde in 100 mm sodium cacodylate, pH 7.2, for 2–3 h at 4°C. Then washed 3x, 10 mins, with 100 mm sodium cacodylate buffer, pH 7.2 at 4°C. They were then post‐fixed with 1% osmium tetroxide in 1.25% potassium ferrocyanide (reduced osmium) for one hour at 4°C, then rinsed 3x with the 100 mm sodium cacodylate buffer, 10 mins each. Rinse 3x with 50 mm sodium maleate buffer, pH 5.2, 10 mins each, and *en bloc* staining with 2.0% uranyl acetate in the maleate buffer overnight at room temperature. Continue with the following steps: rinse 3x with 0.05 m sodium maleate buffer and then proceed with ethyl alcohol dehydration series in the following concentrations: 30%, 50%, 70%, 95%, 100%, 100%. 10 mins each time at room temperature. Following the dehydration series, transition the samples into the following: propylene oxide: 100% ethanol (1:1) for 30 mins, 100% propylene oxide for 30 mins. Finally, into a mixture of propylene oxide: resin (1:1) overnight, on a gentle shaker. Move the samples into propylene oxide: resin (1:2) for the day and then into 100% resin overnight. Finally, place the samples into BEEM capsules and polymerize in the 60°C oven for 48 h. Thin Sections (60 nm) were obtained with a Leica UC7 Ultra‐microtome using a Diatome diamond knife and mounted on carbon‐coated 200 mesh copper grids.

Imaging was performed on a Zeiss Crossbeam 540 scanning electron microscope using an annular STEM detector at an accelerating voltage of 30 kV and probe current of 200pA.

### 4.11 Biochemical Assays

Conditioned media from spheroid cultures were collected every day and stored at −20°C. Human albumin was quantified using an enzyme‐linked immunosorbent assay (ELISA) using a goat anti‐human albumin antibody (Bethyl Laboratories) and 3,3′,5,5′‐tetramethylbenzidine (TMB, Thermo Fisher).

Total bile acid content was quantified from conditioned media through enzymatic cycling of bile acids in the presence of NADH and a chromophore using a commercially available kit (Abcam). Absorbance of the reaction's colorimetric product was read kinetically at 405 nm on a plate reader over time, which could then be converted to a bile acid concentration.

### 4.12 Image Analysis & Quantification

To identify bile canaliculi, ImageJ was used to quantify the CLF signal. A threshold was placed on each image to identify the CLF signal specific to the bile canaliculi (and eliminate the intracellular CLF signal). For each image, the quantity (count) of bile canaliculi was recorded, and for each bile canaliculus, the width and length were calculated using the local thickness and Feret's diameter plugins, respectively. The number of spheroids in each image was quantified manually, and the quantity of bile canaliculi was normalized by the number of spheroids in the image to account for differences in spheroid density across multiple images.

To quantify ductule‐like structures (lined by cholangiocytes), each image was 3D reconstructed using Imaris software (Oxford Instruments). A surface was rendered for the CLF signal for each image, and the surface was segmented using AI image segmentation tool to identify the CLF signal specific to the ductule‐like structures. For each image, the quantity of CLF‐positive ductule‐like structures was recorded, and for each CLF‐positive ductule structure, the volume and sphericity were recorded. The number of spheroids in each image was quantified manually, and the quantity of CLF‐positive ductule‐like structures was normalized by the number of spheroids in the image to account for differences in spheroid density across multiple images.

### 4.13 Spheroid Viability Assay

To measure the viability of spheroids after hypoxia‐reoxygenation conditioning, the CellTiter‐Glo 3D Cell Viability Assay (Promega, Madison, USA) was applied according to the manufacturer's instructions. Briefly, after conditioning, the spheroids were collected from AggreWell plates and seeded into a 96‐well flat white‐bottom plate. A volume of CellTiter‐Glo reagent equal to the volume of the cell culture medium was added and mixed vigorously with the spheroids. The plate was incubated at room temperature for 25 min in the dark. The luminescence was measured by a plate reader (Tecan Infinite M Plex, 30190085).

### 4.14 Statistical Analysis

All statistical analyses were performed using Prism software (GraphPad Software Inc.). Data are presented as the mean ± standard deviation (SD) from at least three replicates. A comparison between two groups was conducted using the unpaired Student's t‐test. Comparisons between multiple groups were performed using a one‐way ANOVA test followed by Tukey's post hoc test. For each test, *p* < 0.05 was considered statistically significant. To indicate statistical significance, one asterisk (^*^) indicates *p* < 0.05, two asterisks (^**^) indicate p < 0.01, three asterisks (^***^) indicate p < 0.001, and four asterisks (^****^) indicate *p* < 0.0001. Exact sample sizes (n), definitions of biological replicates, and any statistical tests used are provided in the corresponding figure legends. Unless otherwise noted, experiments were performed independently twice, and results from one representative experiment are shown.

## Author Contributions

A.D.W., J.K., C.C., and S.N.B. conceived the study; A.D.W., K.A.G., V.K., and B.M.M. designed and performed experiments; A.D.W., K.K., and B.M.M. conducted data analysis; J.Y., J.K., V.K., and A.D.W. established cholangiocyte organoid cultures and contributed technical expertise; D.M., M.E.B., and A.K.R.L. performed scanning transmission electron microscopy; Z.G.J. and D.D.L. contributed key reagents, including primary human liver tissue, and provided clinical expertise; A.D.W., K.A.G., and V.K. wrote the original draft of the manuscript. All authors contributed to manuscript review and editing. C.C. and S.N.B supervised the study and secured funding.

## Funding

A.D.W. acknowledges support from the National Science Foundation Graduate Research Fellowship. Z.G.J. receives research support from NIAA (R01AA030770). The work was supported in part by the Koch Institute Support (core) Grant P30‐CA14051 from the National Cancer Institute, and we thank the Koch Institute's Robert A. Swanson (1969) Biotechnology Center for technical support, specifically the Peterson (1957) Nanotechnology Materials Core Facility (RRID: SCR_018674). This work was supported by the NIH (EB033821) and the Wellcome Leap HOPE Program. S.N.B. is a Howard Hughes Medical Institute Investigator.

## Conflicts of Interest

S.N.B reports compensation for consulting or board membership by Amplifyer Bio, Catalio Capital, Earli Inc., Impilo Therapeutics, Matrisome Bio, Ochre Bio, Port Therapeutics, Ropirio Therapeutics, Satellite Bio, Sunbird Bio, Vertex Pharmaceuticals, and Xilio Therapeutics. All the other authors declare no competing interests.

## Supporting information




**Supporting file 1**: advs75251‐sup‐0001‐Video1.avi.

**Supporting file 2**: advs75251‐sup‐0002‐Video2.avi.

**Supporting file 3**: advs75251‐sup‐0003‐SuppMat.docx.

## Data Availability

The data that support the findings of this study are available from the corresponding author upon reasonable request.
