# Supplementary material for: A 3D In Vitro Model of the Human Hepatobiliary Junction
Source: Adv Sci (Weinh). 2026 Apr 20;13(39):e14855. doi: 10.1002/advs.202514855 (PMC13334853; doi:10.1002/advs.202514855)
Supplement: Supplementary file 3 — Supporting file 3: advs75251‐sup‐0003‐SuppMat.docx. [file ADVS-13-e14855-s001.docx]

**Supplementary Information for:**

**A 3D *in vitro* model of the human hepatobiliary junction**

Authors: **Ashley D. Westerfield^1,2^**, Katarzyna A. Grzelak^1,2^, Katie Katsuyama^3^, Vardhman Kumar^2^, Bess M. Miller^2^, Joa Yun^2^, Jesse Kirkpatrick^1,2,6^, David Mankus^2^, Margaret E. Bisher^2^, Abigail K.R. Lytton-Jean^2^, Z. Gordon Jiang^7^, David D. Lee^8^, Christopher S. Chen^9,10^, Sangeeta N. Bhatia^1,2,4-6,10,11*^

Affiliations:

1. Institute of Medical Engineering and Science, Massachusetts Institute of Technology, Cambridge, MA 02139, USA
2. David H. Koch Institute for Integrative Cancer Research, Massachusetts Institute of Technology, Cambridge, MA 02139, USA
3. Department of Biological Engineering, Massachusetts Institute of Technology, Cambridge, MA 02139, USA
4. Department of Electrical Engineering and Computer Science, Massachusetts Institute of Technology, Cambridge, MA 02139, USA
5. Broad Institute of MIT and Harvard, Cambridge, MA 02139, USA
6. Department of Medicine, Brigham and Women’s Hospital, Boston, MA 02115, USA
7. Department of Medicine, Beth Israel Deaconess Medical Center, Boston, MA 02215, USA
8. Department of Surgery, Beth Israel Deaconess Medical Center, Boston, MA 02215, USA
9. Department of Biomedical Engineering and the Biological Design Center, Boston University, Boston, MA 02215, USA
10. Wyss Institute for Biologically Inspired Engineering at Harvard University, Boston, MA 02215, USA
11. Howard Hughes Medical Institute, Chevy Chase, MD 20815, USA

^*^Corresponding author. Email: sbhatia@mit.edu


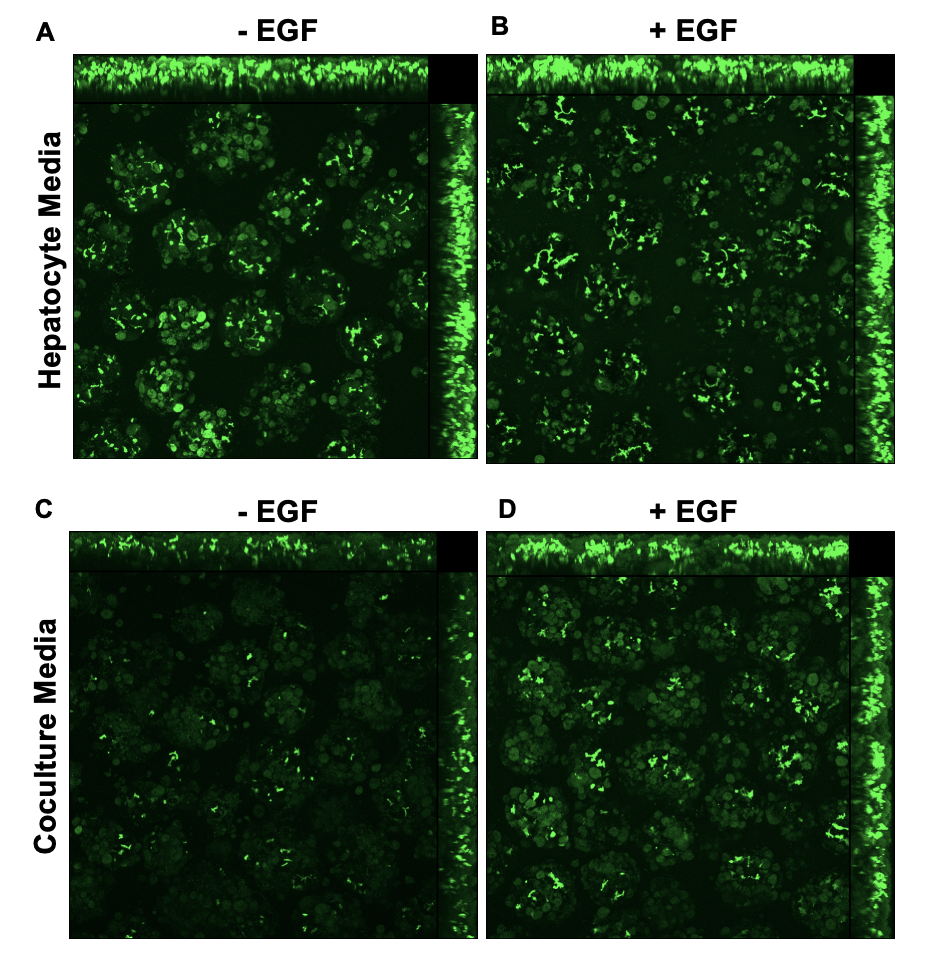


Supplementary Figure 1: EGF improves bile canaliculi formation in biaggregate spheroids in two different media conditions. Representative images of CLF assay on biaggregate spheroids in hepatocyte media without (A) and with (B) 50ng/ml EGF, and in coculture media without (C) and with (D) 50ng/ml EGF. Shown as descriptive optimization; statistical comparisons were not performed.


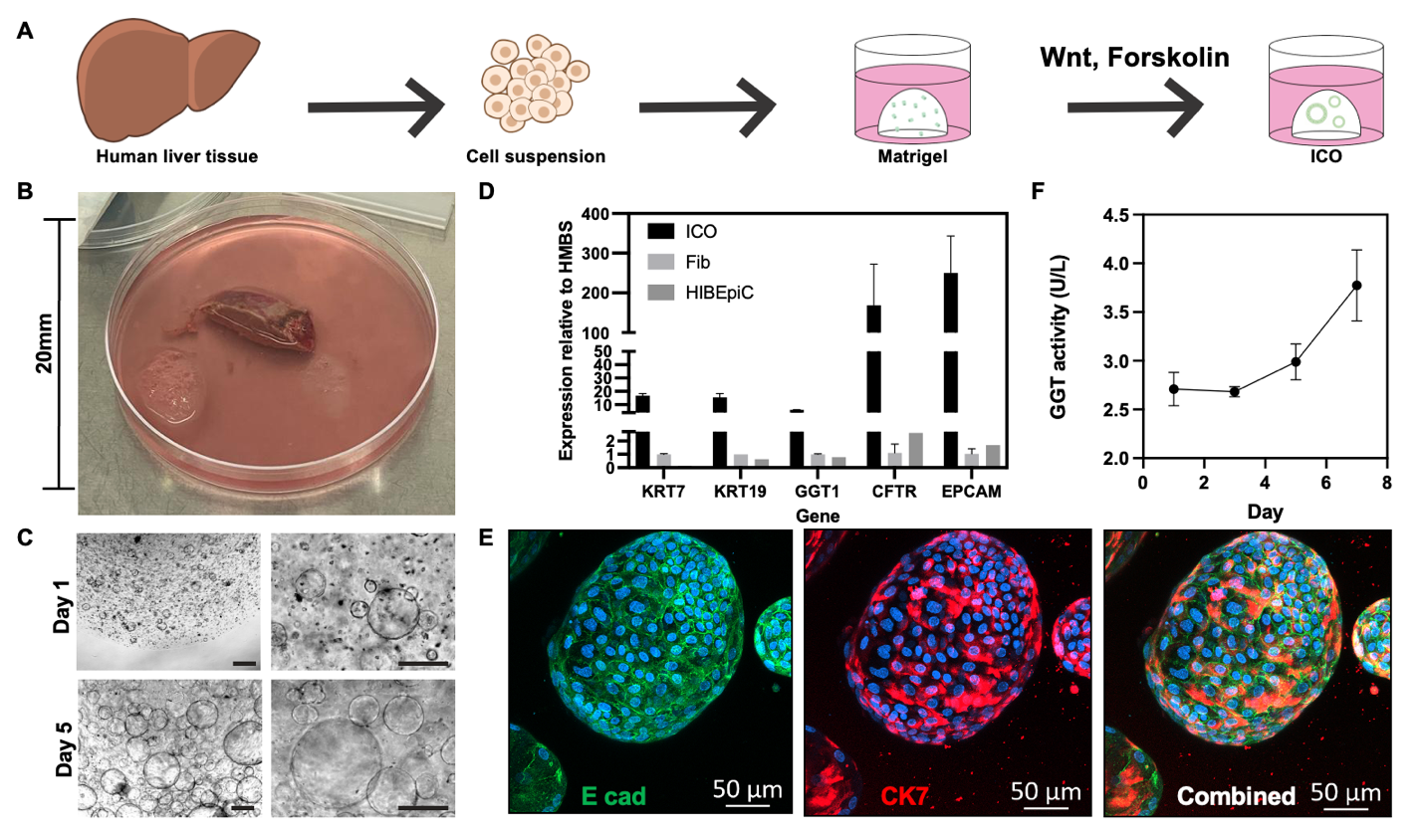


Supplementary Figure 2: Establishing an intrahepatic cholangiocyte organoid (ICO) line from adult human liver biopsy tissue

1. Schematic depicting protocol used to isolate and culture cholangiocytes as organoids from an adult human liver biopsy tissue.
2. Macroscopic image of human liver biopsy tissue (~4mm) in a 20mm Petri dish.
3. Brightfield microscope images of ICO in Matrigel culture, 1 day (top) and 5 days (bottom) after first passage in culture, scale bar = 500 μm.
4. Gene expression of cholangiocyte specific genes in ICO culture, detected by RT-qPCR. Also graphed are gene expression levels in primary human fibroblasts (Fib) and a commercially-available cholangiocyte cell line (HIBEpiC). Data are shown as mean ± SD with individual data points representing independent wells (n = 3).
5. Immunofluorescence staining of E cadherin (green) and CK7 (red) in ICOs, scale bar = 50 μm.
6. GGT activity measured in media collected from ICO culture. Data are shown as mean ± SD with individual data points representing independent wells (n = 3).


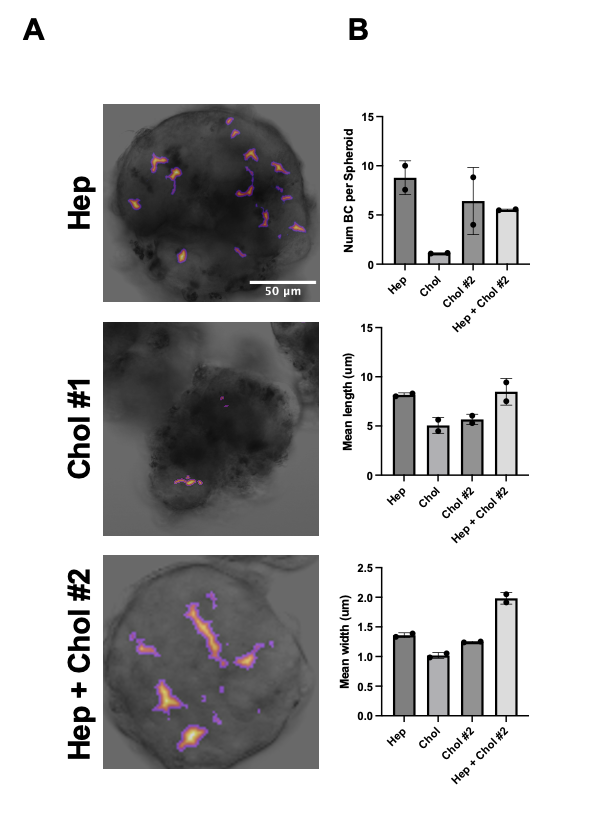


Supplementary Figure 3: Media composition plays a significant role in bile canalicular secretion of CLF in biaggregate spheroids.

1. Representative images width-coded for CLF signal in hepatocyte media (top), cholangiocyte media (middle), and optimized coculture media (bottom).
2. Quantification of number (top), length (middle), and width (bottom) of bile canaliculi in different media conditions. Data are shown as mean ± SD with individual data points representing independent wells (n = 2). Shown as descriptive optimization; statistical comparisons were not performed.


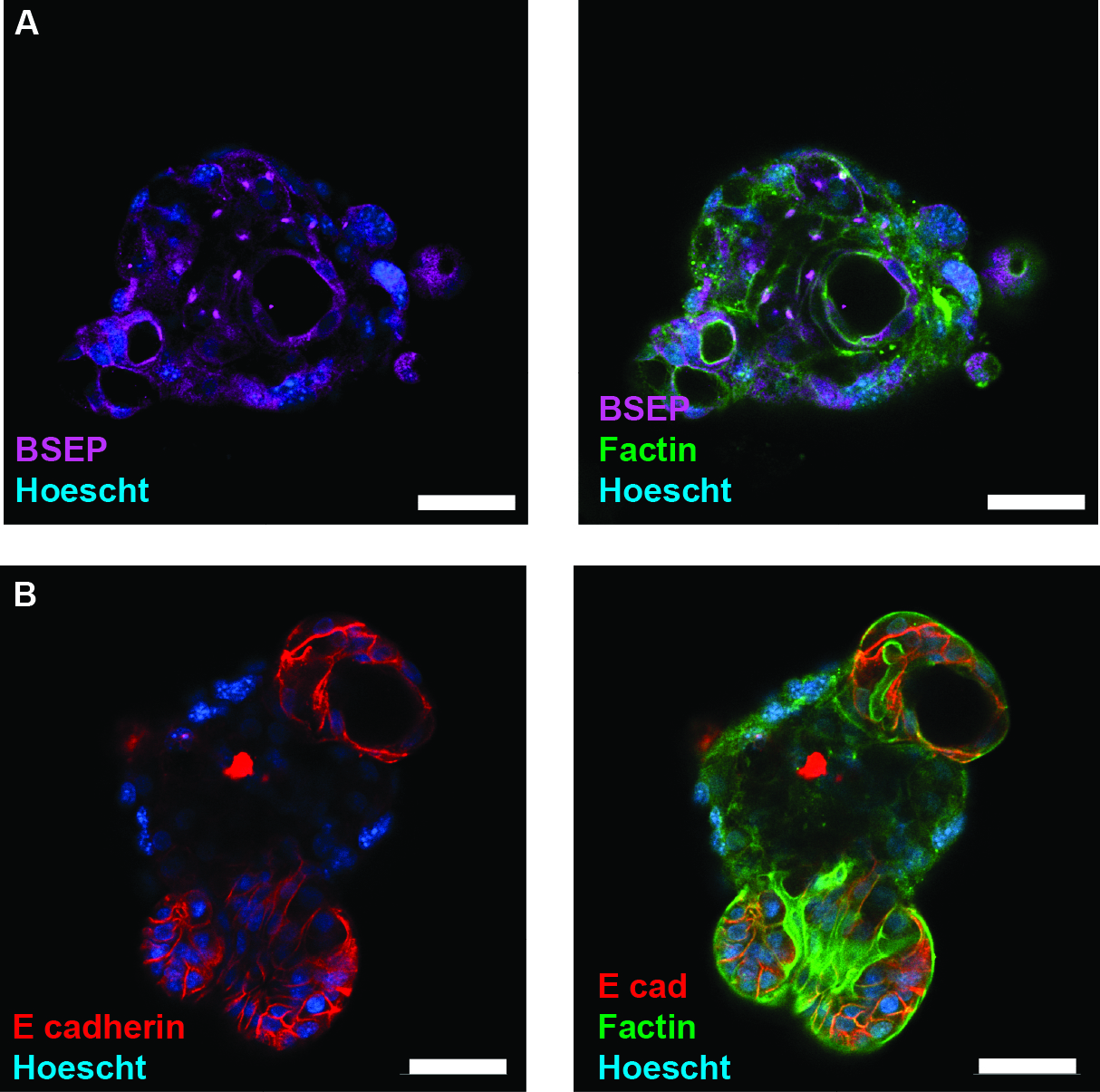


Supplementary Figure 4: Protein expression and localization of BSEP (A), and E cadherin (B), with and without F-actin (A and B, right, in green) in 3D confocal microscope images of aHBOs after 3 days in culture, scale bars = 50 μm.

| Donor | CLF | Brightfield |
| --- | --- | --- |
| Lonza, HUM190131 | 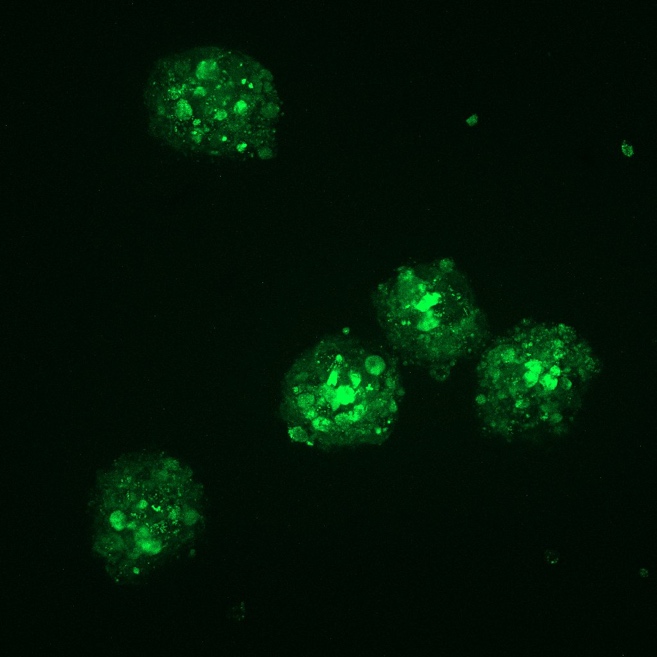 | 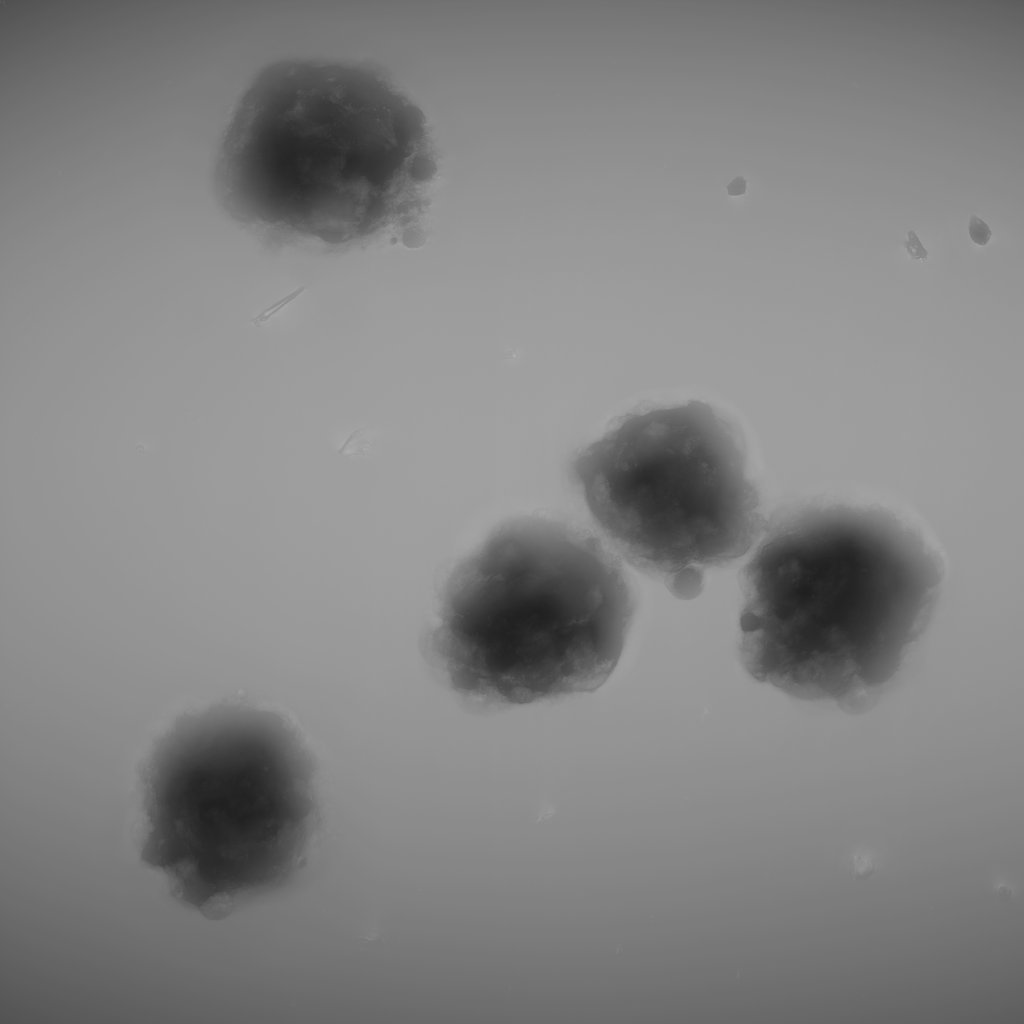 |
| Lonza, HUM183001 | 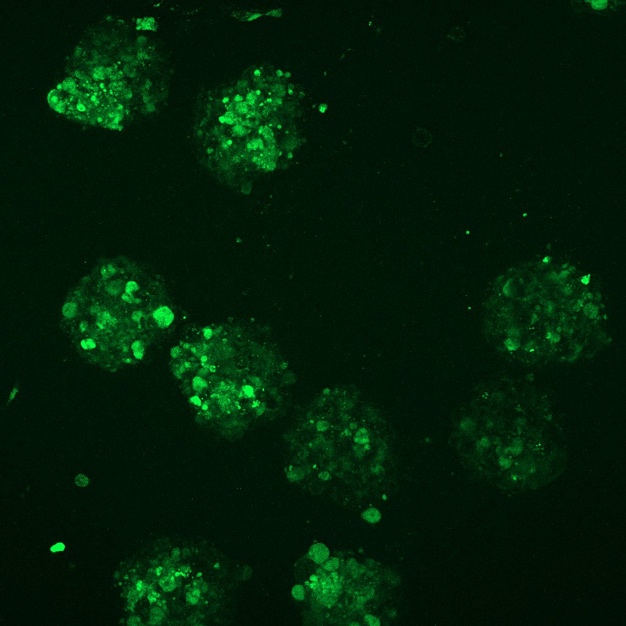 | 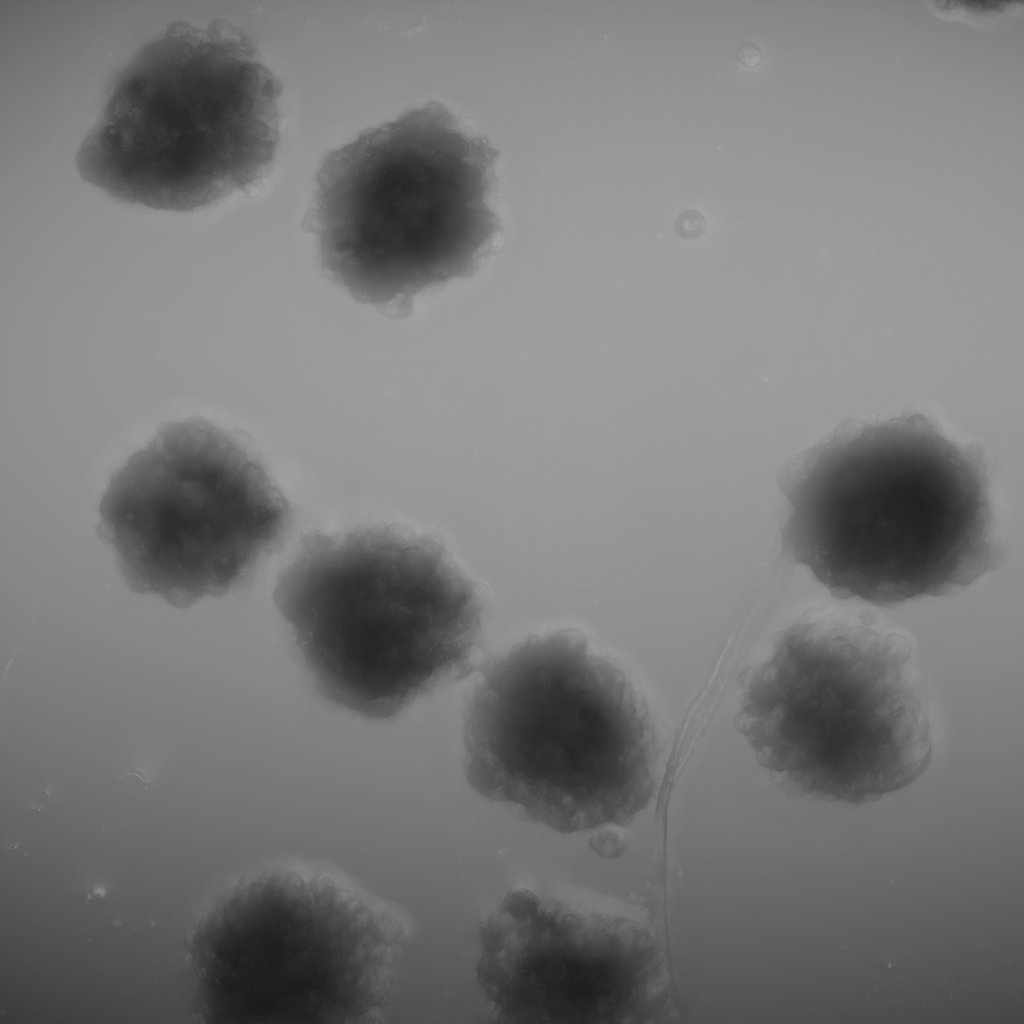 |
| Lonza, HUM200691 | 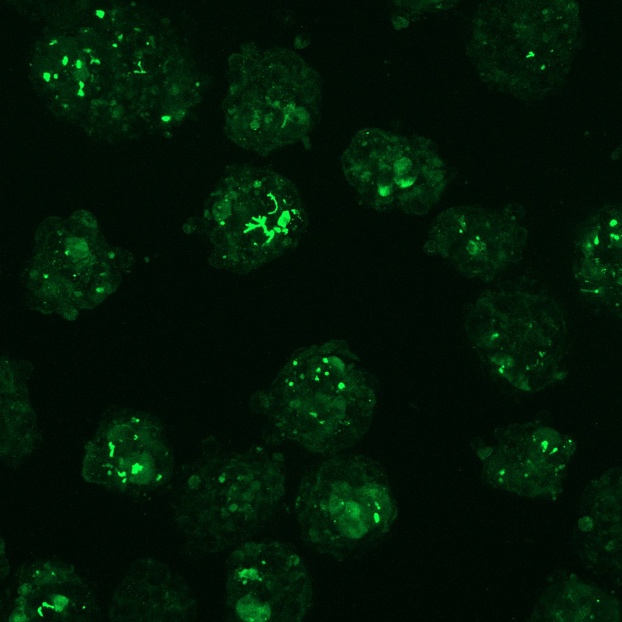 | 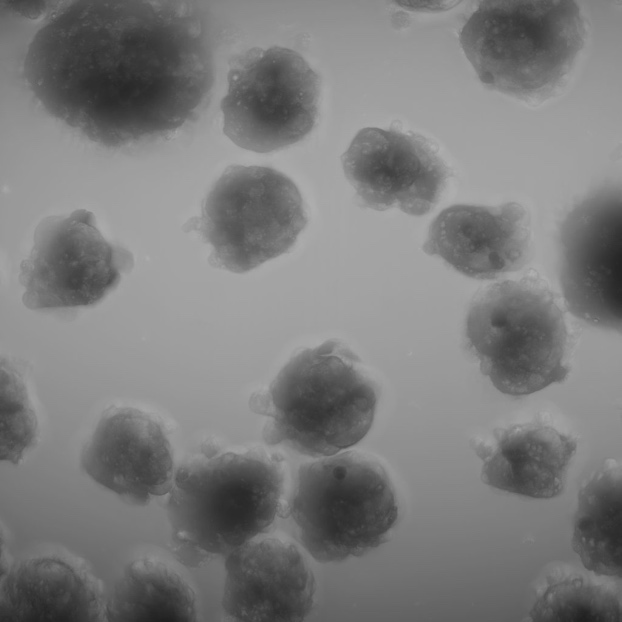 |
| Lonza, HUM221971 | 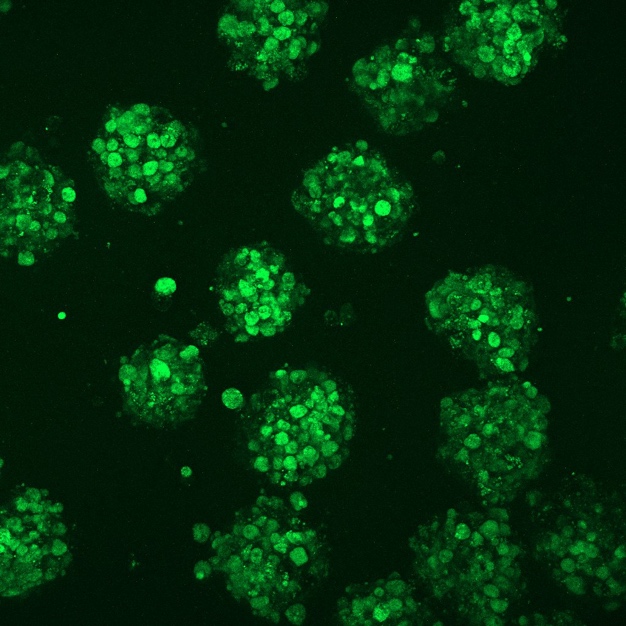 | 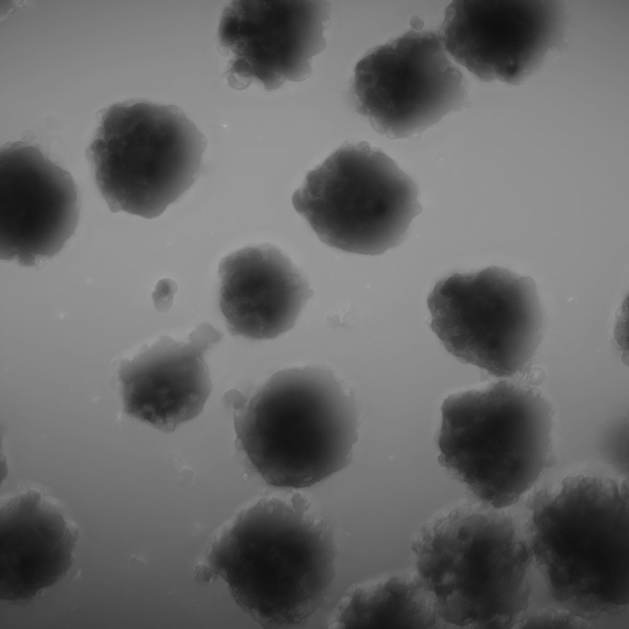 |
| Lonza, HUM222051 | 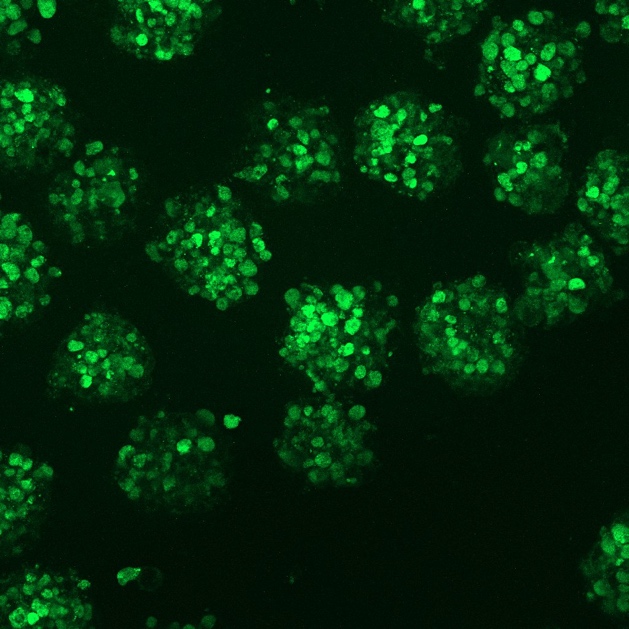 | 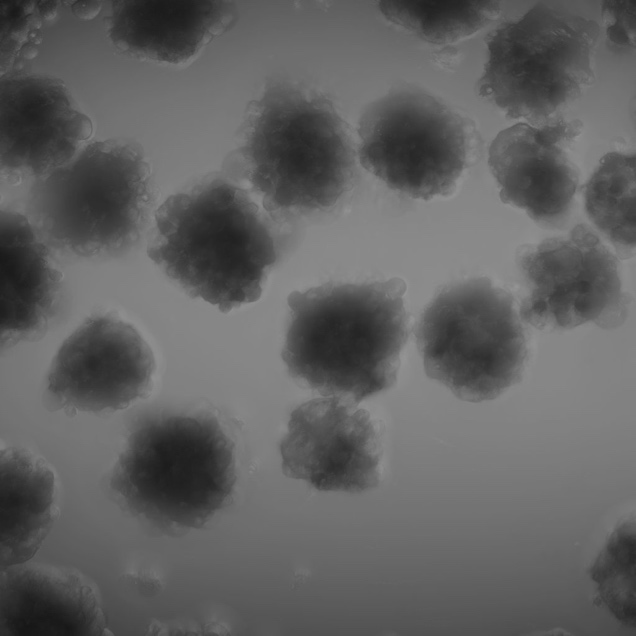 |
| Lonza, HUM182851 | 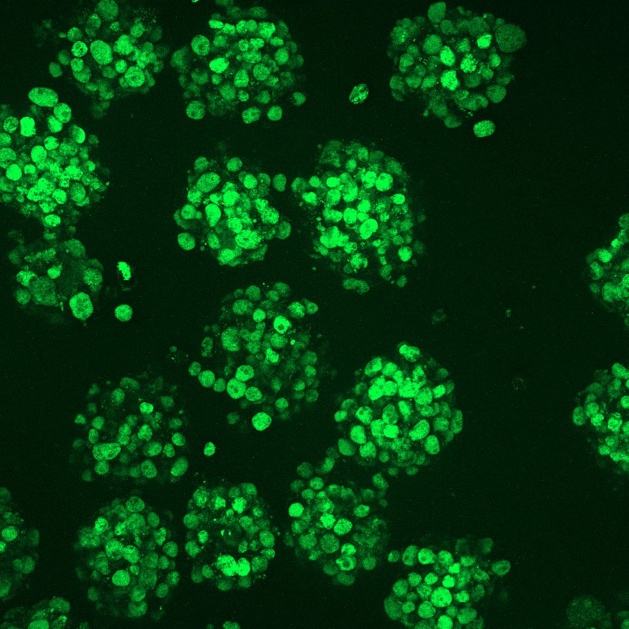 | 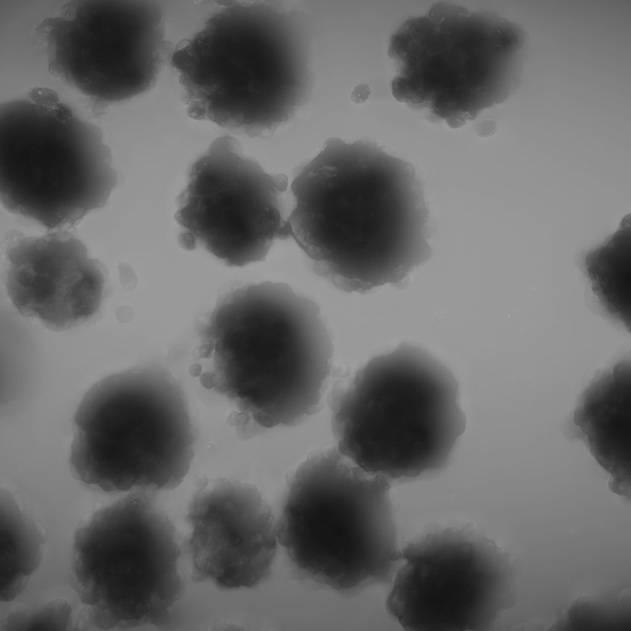 |
| Lonza, HUM182531 | 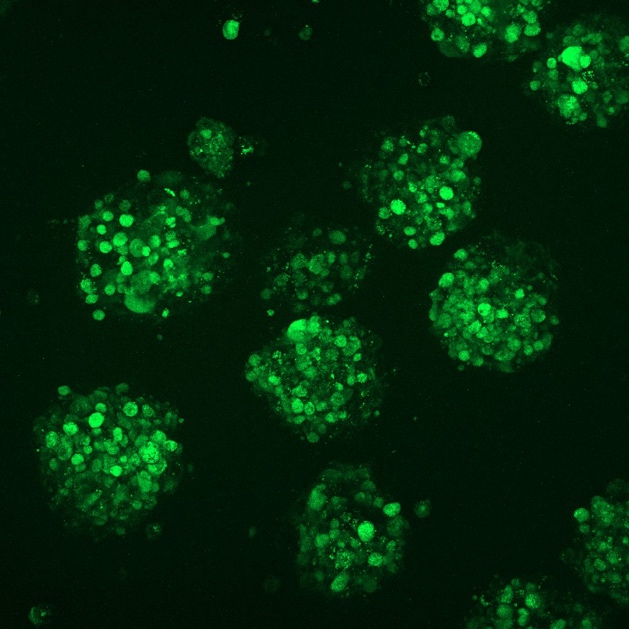 | 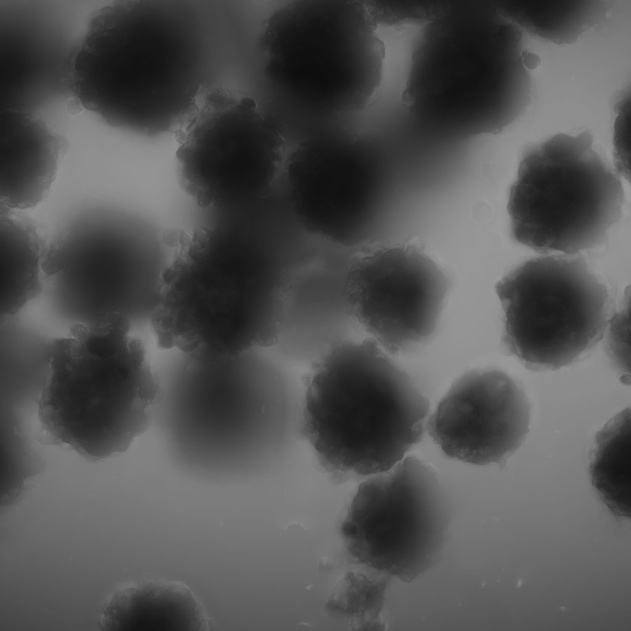 |
| Lonza, HUM183121 | 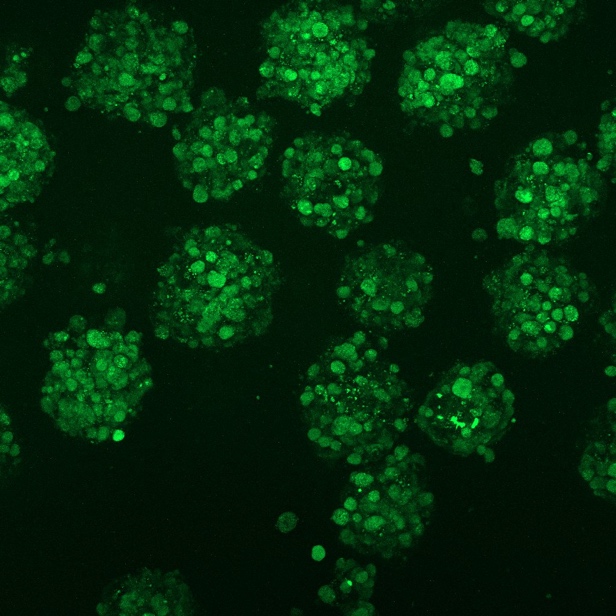 | 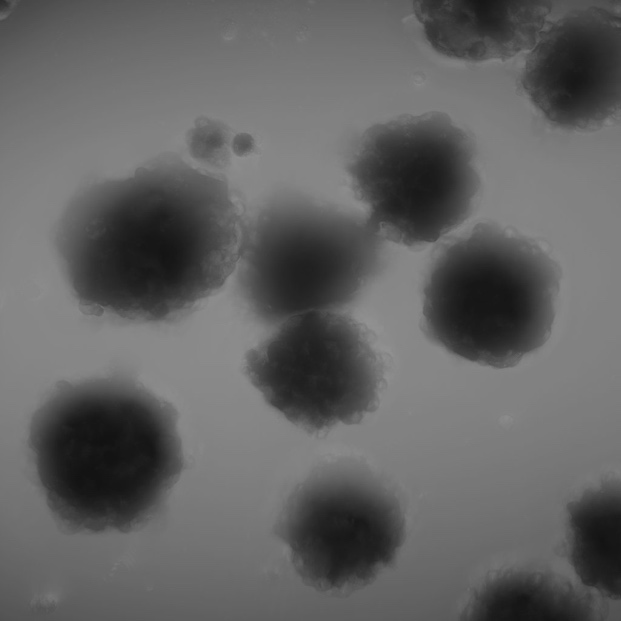 |
| Thermo, HU8406 | 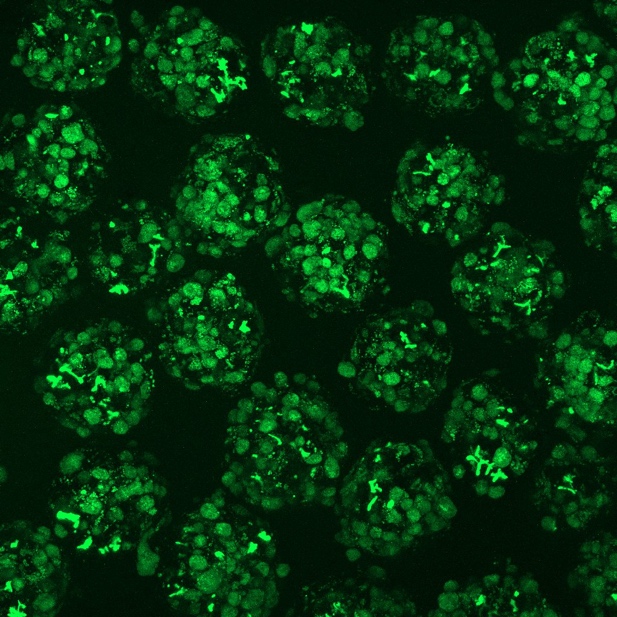 | 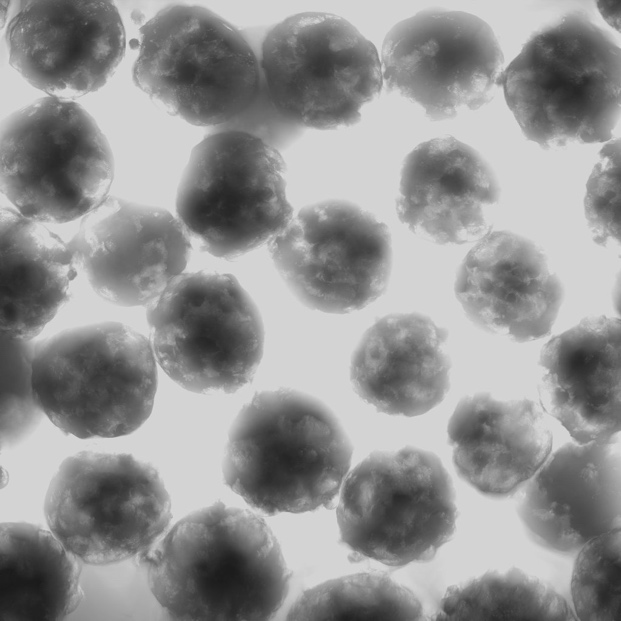 |
| Thermo, HU8412 | 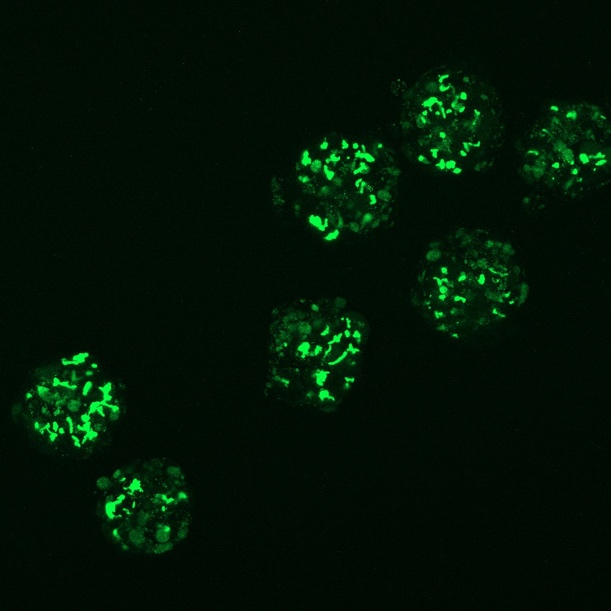 | 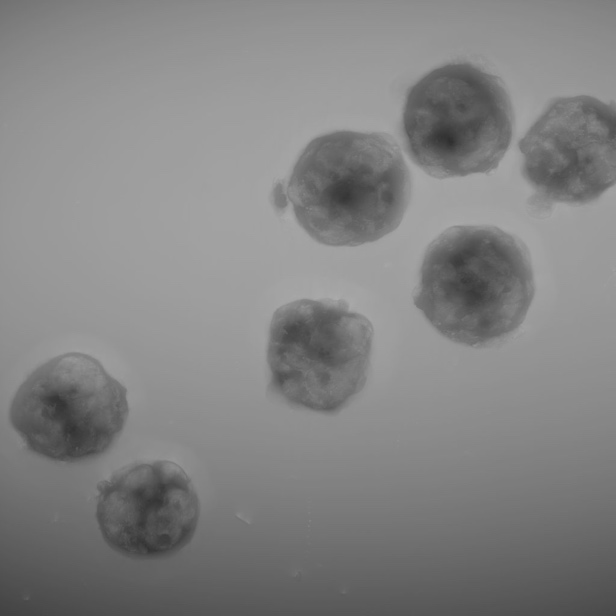 |
| Thermo, HU8444 | 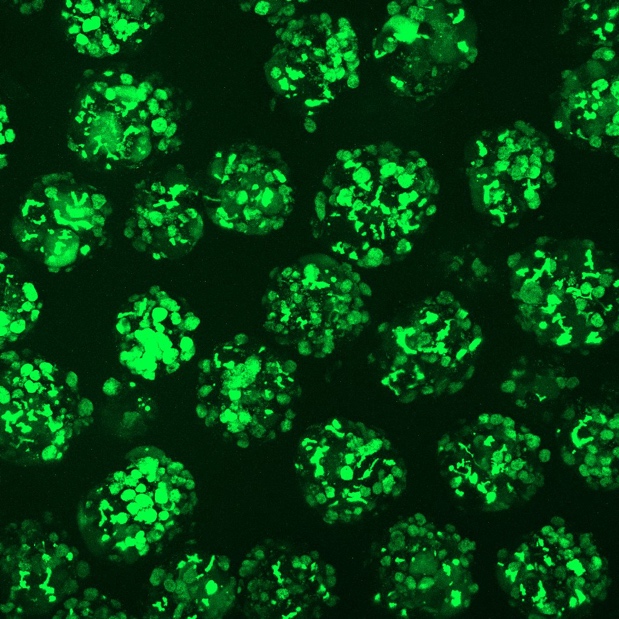 | 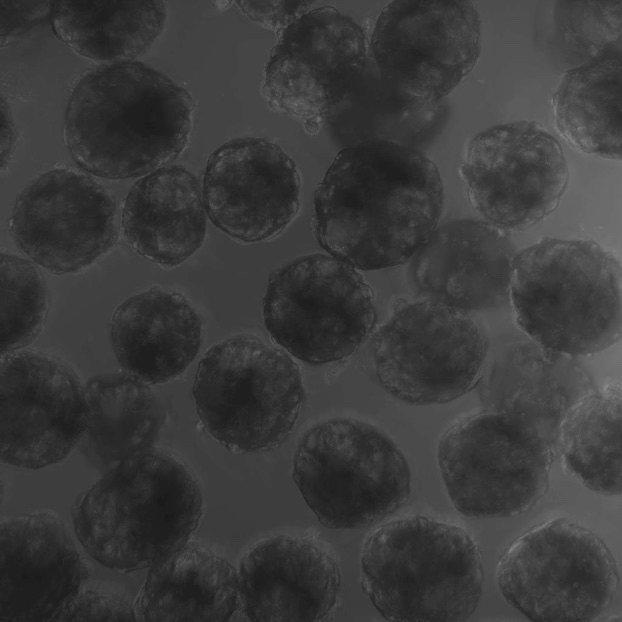 |
| Thermo, HU8447 | 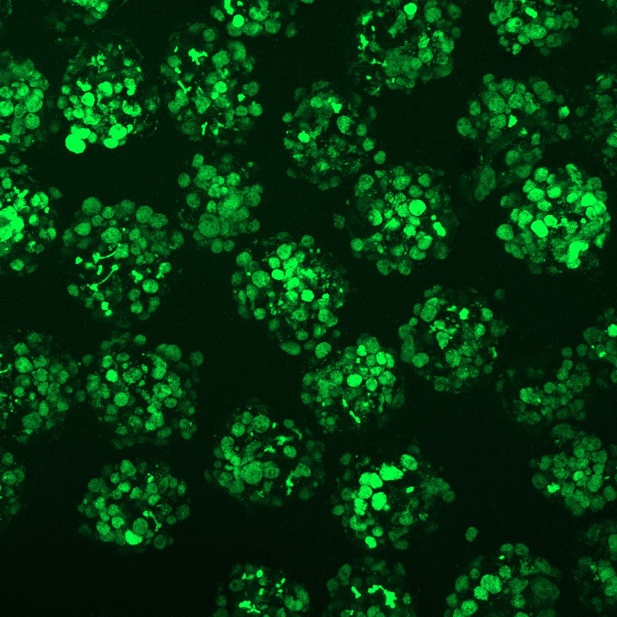 | 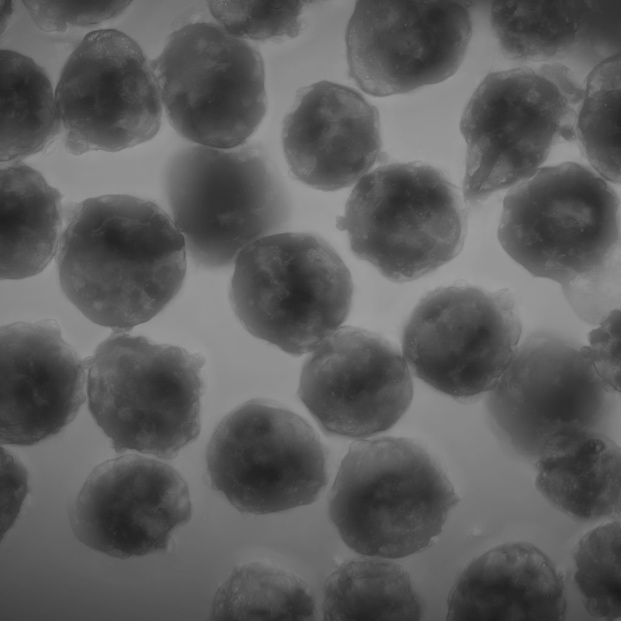 |
| Thermo, HU8449 | 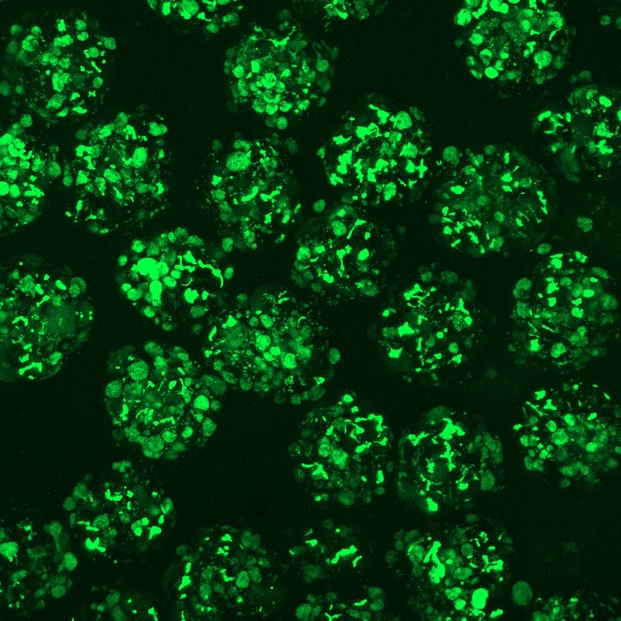 | 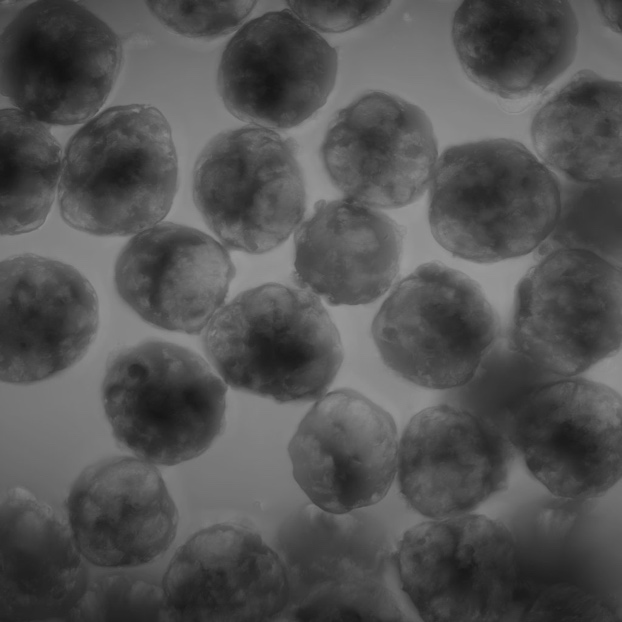 |
| Thermo, HU8452 | 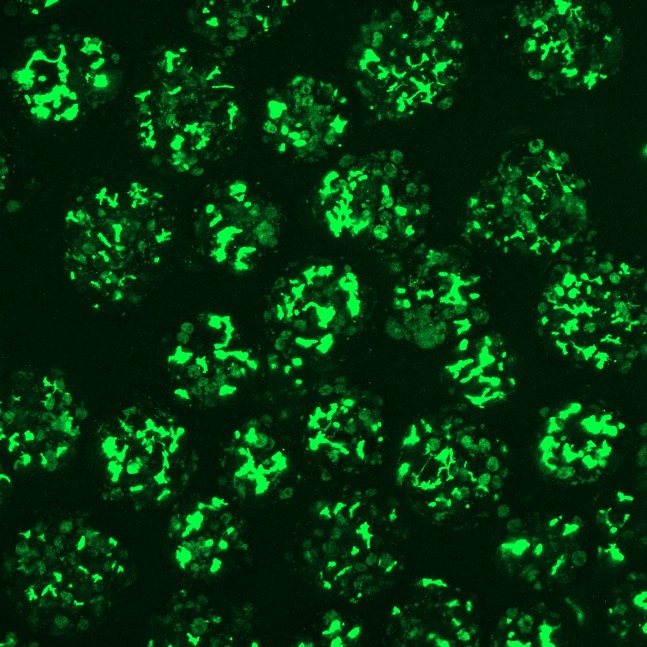 | 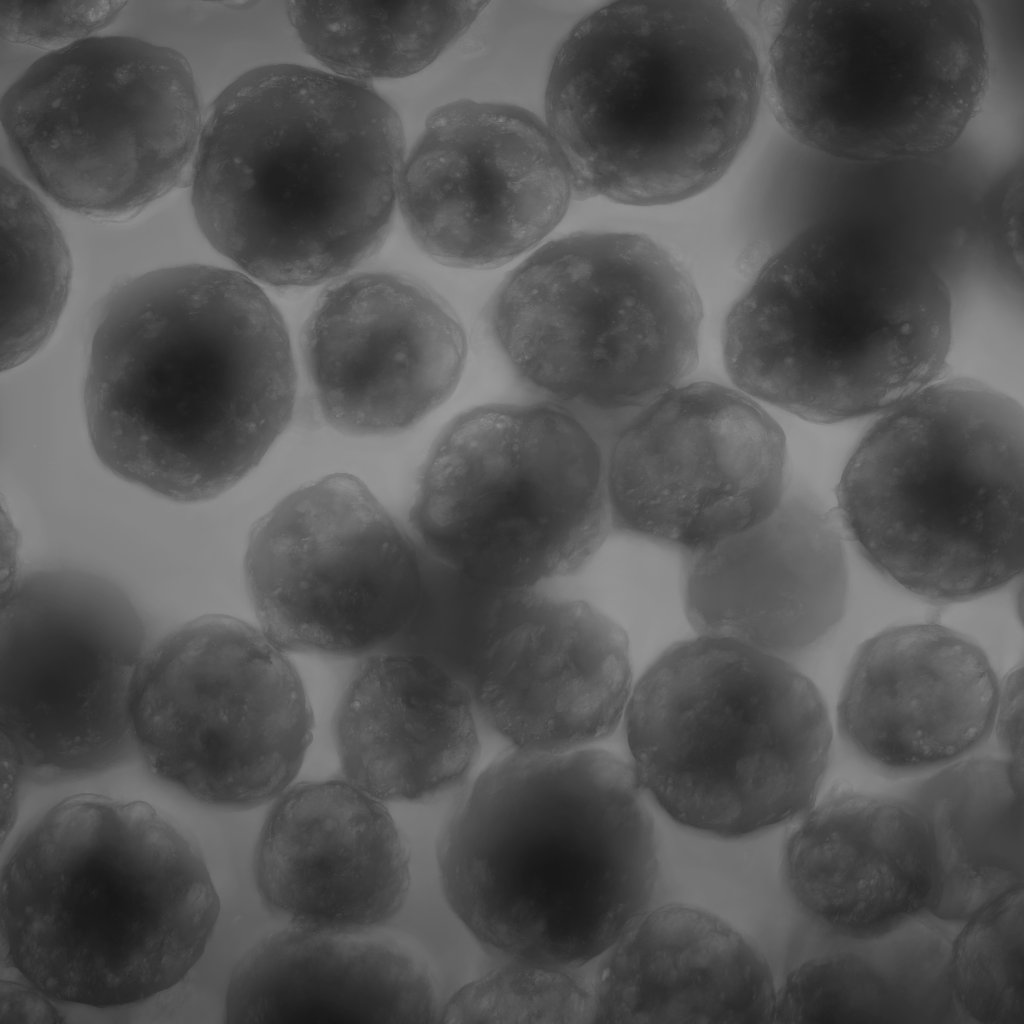 |
| Thermo, 8200 | 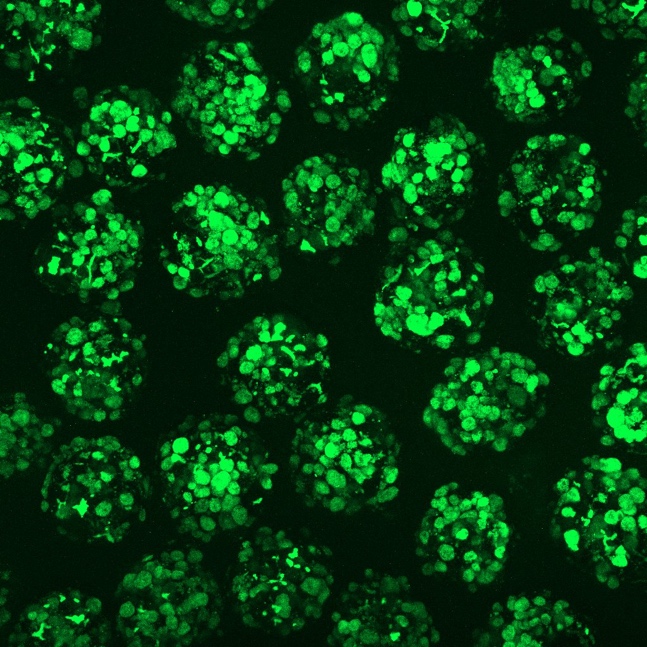 | 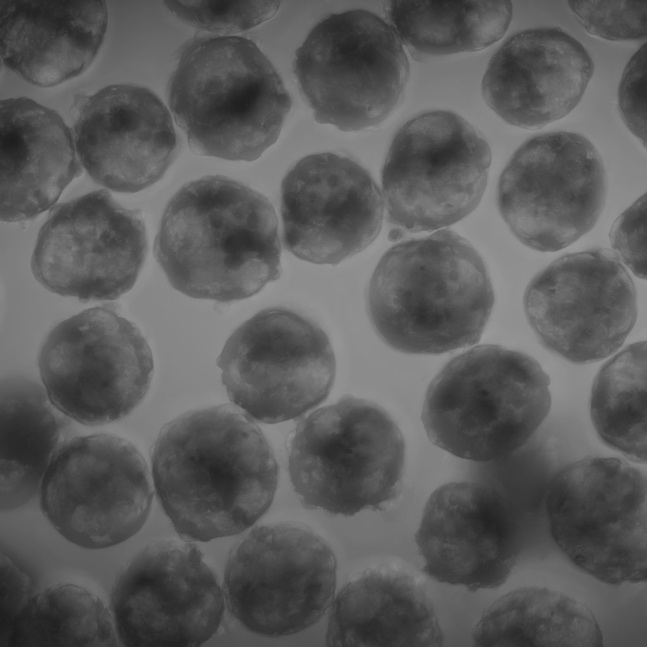 |
| Thermo, 4129 | 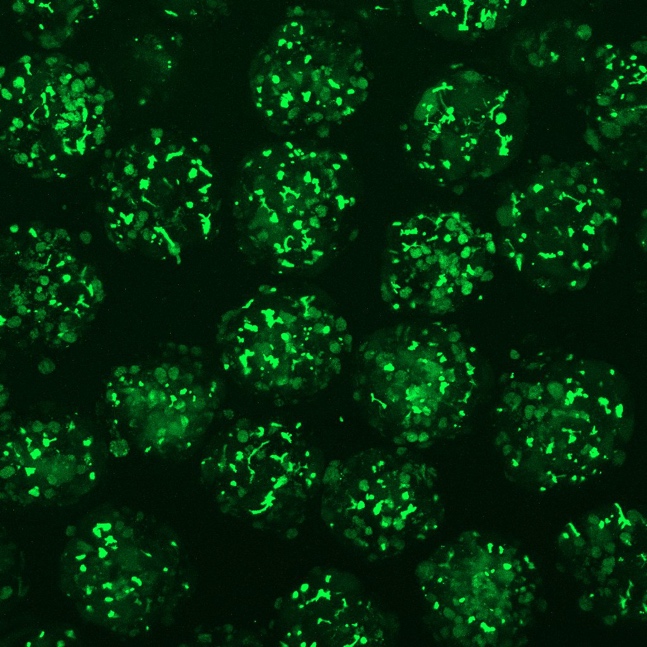 | 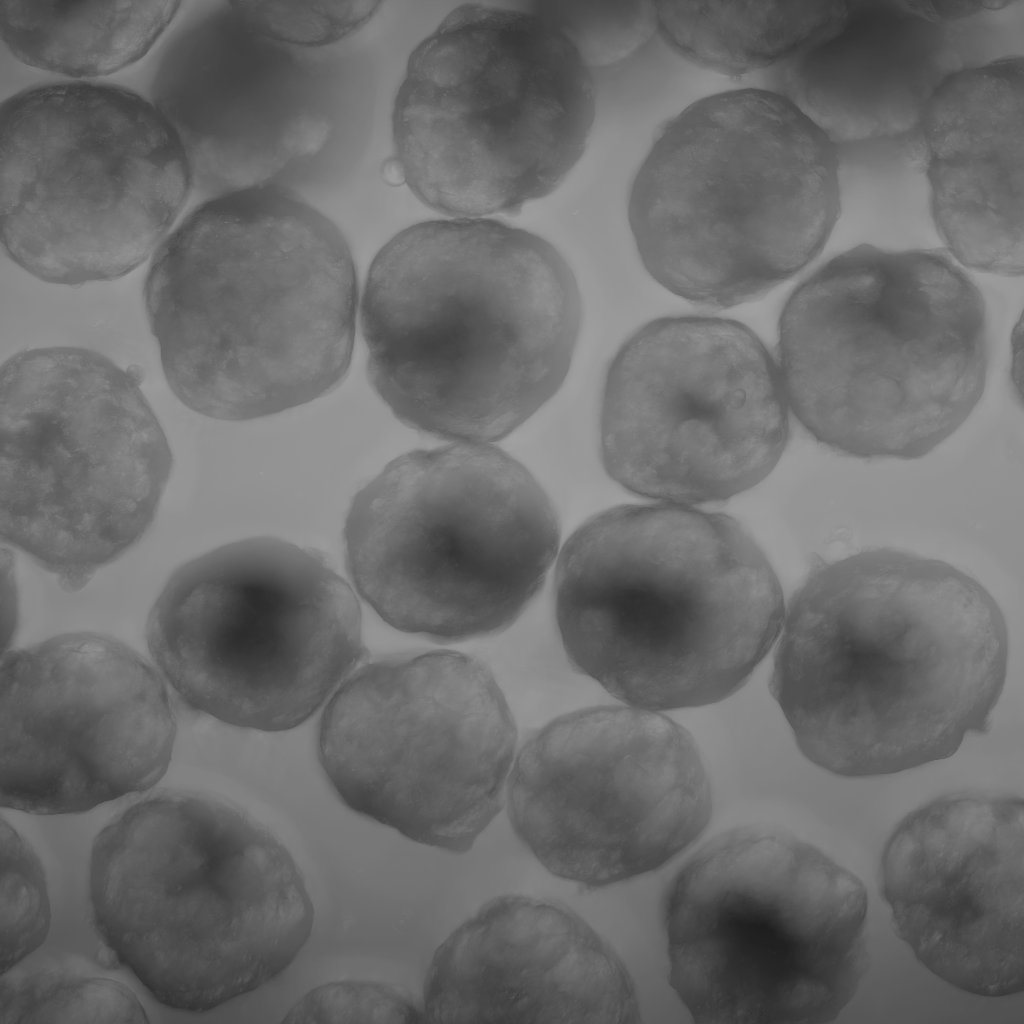 |
| BioIVT, ZGF | 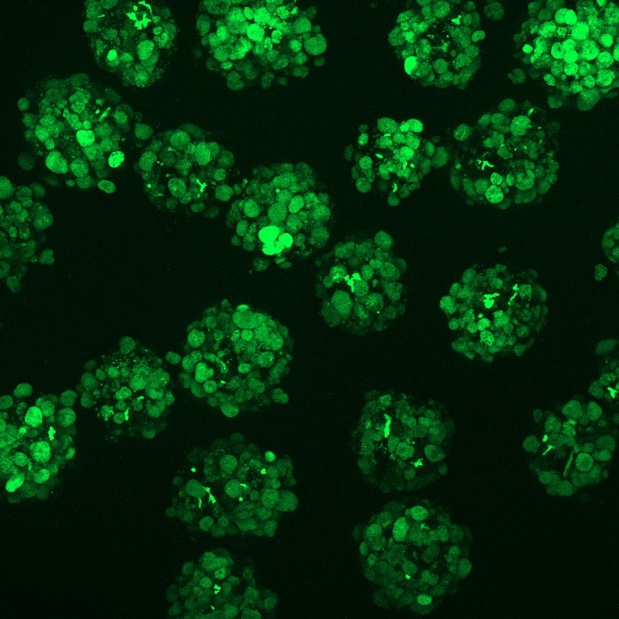 | 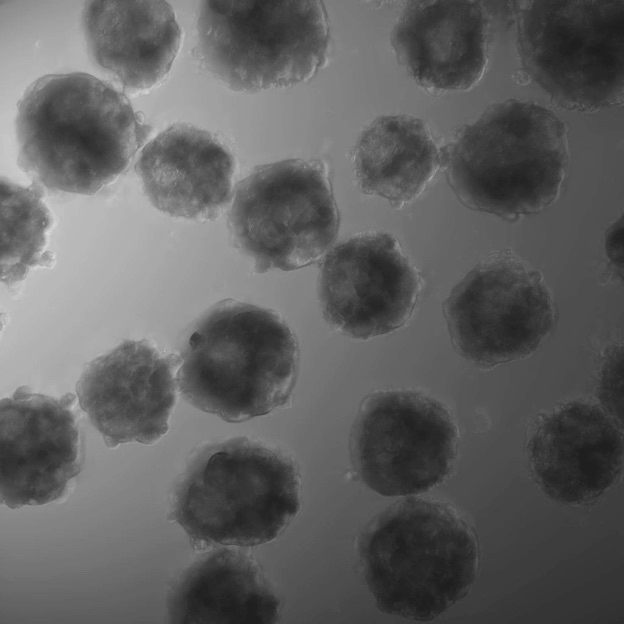 |

Supplementary Figure 5: Representative CLF and brightfield images of primary hepatocyte spheroids generated from 17 human donors from 3 different commercial vendors.

Table S1: Media Formulations

| Hepatocyte Media | | |
| --- | --- | --- |
| **Reagent** | **Vendor + Cat No.** | **Concentration** |
| DMEM with L-glutamine | Corning, 10-017-CV | N/A |
| ITS+ Premix Universal Culture Supplement | Corning, 354352 | 1% |
| Fetal bovine serum | GeminiBio | 10% |
| Dexamethasone | R&D Systems, 1126/100 | 0.04 ug/ml |
| Glucagon | Sigma-Aldrich, G2044-1MG | 7 ng/ml |
| HEPES | Sigma-Aldrich, H0887-20ML | 15mM |
| Penicillin/streptomycin | Life Technologies, 15140122 | 1% |
|  |  |  |
| ICO expansion media | | |
| **Reagent** | **Vendor + Cat No.** | **Concentration** |
| B27 Supplement 50x, minus vitamin A | Life Technologies, 12587-010 | 1x |
| N2 supplement 100x | ﻿Life Technologies, 17502-048 | 1x |
| N-acetylcysteine | ﻿Sigma-Aldrich, A0737-5MG | 1mM |
| Recombinant human [Leu15]-gastrin I | Sigma-Aldrich, G9145 | 10nM |
| Recombinant human EGF | ﻿Peprotech, AF-100-15 | 50ng/ml |
| Rspo1-conditioned medium | Made in house | 10% |
| Recombinant human FGF10 | ﻿Peprotech, 100-26 | 100ng/ml |
| Recombinant human HGF | ﻿Peprotech, 100-39 | 25ng/ml |
| Nicotinamide | ﻿Sigma-Aldrich, N0636 | 10mM |
| A83-01 | ﻿Tocris Bioscience, 2939 | 5uM |
| Forskolin | Enzo, BML-CN100-0010 | 10uM |
| Advanced DMEM/F12 | Thermo Fisher, 12634010 | N/A |
|  | | |
| Cholangiocyte media | | |
| **Reagent** | **Vendor + Cat No.** | **Concentration** |
| Nicotinamide | Sigma-Aldrich, N0636 | 10mM |
| Sodium bicarbonate | Sigma-Aldrich, S5761 | 17mM |
| 2-phospho-L-ascorbic acid trisodium salt | Sigma-Aldrich, 49752 | 200uM |
| Sodium pyruvate | Thermo Fisher, 11360070 | 0.63mM |
| Glucose | Invitrogen, 15023021 | 14mM |
| HEPES | Sigma-Aldrich, H0887-20ML | 20mM |
| ITS+ Premix Universal Culture Supplement | Corning, 354352 | 1% |
| Dexamethasone | R&D Systems, 1126/100 | 0.1uM |
| Glutamax | Lonza, BE17-605E/U1 | 1% |
| Penicillin/streptomycin | Life Technologies, 15140122 | 1% |
| Recombinant human EGF | ﻿Peprotech, AF-100-15 | 20ng/ml |
| Rspo1-conditioned medium | Made in house [16] | 10% |
| Recombinant human DKK1 | Abcam, ab155623 | 100ng/ml |
| William’s E Medium, no phenol red | Invitrogen, A1217601 | N/A |

Table S2: List of qPCR primer sequences

| **Gene Name** | **Forward Primer** | **Reverse Primer** |
| --- | --- | --- |
| HMBS | ACGGCTCAGATAGCATACAAGAG | GTTACGAGCAGTGATGCCTACC |
| BSEP | AGCCACACAGACCAGGATGTTG | CAATGAACCGCCTCTCCTTTCC |
| MRP2 | GCCAACTTGTGGCTGTGATAGG | ATCCAGGACTGCTGTGGGACAT |
| NTCP | GCTCTCTTCTGCCTCAATGGAC | AGTGGTCCAATGACTTCAGGTGG |
| CDH1 | GCCTCCTGAAAAGAGAGTGGAAG | TGGCAGTGTCTCTCCAAATCCG |
| CYP7A1 | CAAGCAAACACCATTCCAGCGAC | ATAGGATTGCCTTCCAAGCTGAC |
| KRT7 | TGTGGATGCTGCCTACATGAGC | AGCACCACAGATGTGTCGGAGA |
| KRT19 | AGCTAGAGGTGAAGATCCGCGA | GCAGGACAATCCTGGAGTTCTC |
| GGT1 | TGACGTACCACCGCATCGTAGA | CAGCGAAGAACTCGGAGGTCAT |
| CFTR | GGAGAGCATACCAGCAGTGACT | TTCCAAGGAGCCACAGCACAAC |
| EPCAM | GCCAGTGTACTTCAGTTGGTGC | CCCTTCAGGTTTTGCTCTTCTCC |
